# A Comprehensive Understanding of Postural Tone Biomechanics: Intrinsic Stiffness, Functional Stiffness, Antagonist Coactivation, and COP Dynamics in Post-Stroke Adults

**DOI:** 10.3390/s25072196

**Published:** 2025-03-30

**Authors:** Liliana Pinho, Marta Freitas, Francisco Pinho, Sandra Silva, Vânia Figueira, Edgar Ribeiro, Andreia S. P. Sousa, Filipa Sousa, Augusta Silva

**Affiliations:** 1 Escola Superior de Saúde do Vale do Ave, Cooperativa de Ensino Superior Politécnico e Universitário, Rua José António Vidal, 81, 4760-409 Vila Nova de Famalicão, Portugal; liliana.pinho@ipsn.cespu.pt (L.P.); marta.goncalves@ipsn.cespu.pt (M.F.); sandra.silva@ipsn.cespu.pt (S.S.); vania.figueira@ipsn.cespu.pt (V.F.); 2 Centre of Research Rehabilitation (CIR), Escola Superior de Saúde, rua Dr. António Bernardino de Almeida 400, 4200-072 Porto, Portugal; edgaroribeiro@gmail.com (E.R.); asp@ess.ipp.pt (A.S.P.S.); augusta.silva@ess.ipp.pt (A.S.); 3 Faculty of Sports, University of Porto, 4200-450 Porto, Portugal; 4 H2M—Health and Human Movement Unit, Polytechnic University of Health, Cooperativa de Ensino Superior Politécnico e Universitário, Cooperativa de Responsabilidade Limitada, 4760-409 Vila Nova de Famalicão, Portugal; 5 Department of Medical Sciences, University of Aveiro, 3810-193 Aveiro, Portugal; 6 School of Health Sciences, University of Aveiro, 3810-193 Aveiro, Portugal; 7 Centre for Research, Education, Innovation, and Intervention in Sport (CIFI2D), Faculty of Sport of the University of Porto (FADEUP), 4050-313 Porto, Portugal; filipas@fade.up.pt; 8 Laboratory of Biomechanics, University of Porto, 4050-313 Porto, Portugal

**Keywords:** muscle tone, postural control, neuromuscular adaptation, non-linear analysis, biomechanical stability

## Abstract

Objective: To analyse the relationship between traditional stiffness and muscle antagonist coactivation in both stroke and healthy participants, using linear and non-linear measures of coactivation and COP during standing, stand-to-sit, and gait initiation. Methods: Participants were evaluated through a cross-sectional design. Electromyography, isokinetic dynamometer, and force plate were used to calculate coactivation, intrinsic and functional stiffness, and COP displacement, with both linear and non-linear metrics. Spearman’s correlations and Mann–Whitney tests were applied (*p* < 0.05). Results: Post-stroke participants showed higher contralesional intrinsic stiffness (*p* = 0.041) and higher functional stiffness (*p* = 0.047). Coactivation was higher on the ipsilesional side during standing (*p* = 0.012) and reduced on the contralesional side during standing and transitions (*p* < 0.01). Moderate correlations were found between intrinsic and functional stiffness (*p* = 0.030) and between coactivation and intrinsic stiffness (standing and stand-to-sit: *p* = 0.048) and functional stiffness (gait initiation: *p* = 0.045). COP displacement was reduced in post-stroke participants during standing (*p* < 0.001) and increased during gait initiation (*p* = 0.001). Post-stroke participants exhibited increased gastrocnemius/tibialis anterior coactivation during gait initiation (*p* = 0.038) and higher entropy and stability across tasks (*p* < 0.001). Conclusion: Post-stroke participants showed higher contralesional intrinsic and functional stiffness, reduced coactivation in static tasks, and increased coactivation in dynamic tasks. COP and coactivation analyses revealed impaired stability and random control, highlighting the importance of multidimensional evaluations of postural tone.

## 1. Introduction

As described by Nikolai Bernstein, muscle tone is a complex and dynamic condition that underpins the central nervous system’s (CNS) functional adaptations [1,2]. It arises from hierarchical and reciprocal anatomical and neurological connectivity, representing a state of readiness for movement [3], and is regulated through intricate interactions between neural input and output systems, adapting to meet task-specific performance and biomechanical requirements [4]. Fundamentally, it acts as a construct of motor control, balancing force, coordination, and task execution [2].

While traditional approaches provide valuable insights into certain aspects of muscle tone, a more comprehensive evaluation is needed to fully capture its neurofunctional and biomechanical dimensions [4]. Methods such as asking a person to relax without performing any movement tend to focus on its mechanical properties, such as passive stiffness, which characterises the deformability of certain bodies under the influence of external forces [4,5,6]. Although informative, these methods may not fully reflect the active neural and sensory components of tone. Bernstein’s hierarchical model further emphasises that muscle tone reflects a state of preparedness for movement, integrating sensory feedback and motor commands to enable both postural and movement control [4]. Thus, to structure a logical progression of the literature, it is important to consider how different models and methodologies have evolved to improve our understanding of muscle tone. Early models primarily focused on mechanical properties such as passive stiffness [3,7], which provided insight into the elasticity and resistance of muscles but failed to capture active neuromuscular components. Subsequent models integrated motor control theories, emphasising the role of sensory feedback and neuromuscular adaptation [3,4,8]. Given this emerging dynamic understanding, there is a growing need for complementary approaches that capture both static and dynamic aspects of muscle tone.

Increased passive stiffness, a measurable component of muscle tone, has been identified in post-stroke patients [3,7], particularly in distal segments such as the plantar flexors [9,10]. However, passive stiffness alone fails to capture the active components of tone, which depend on the integration of neuronal circuits, sensory feedback, and dynamic motor strategies [3,4,11]. Complementing this perspective, a broader and more dynamic approach of tone—postural tone—also reflects tonic muscle activation that supports antigravity functions and facilitates the continuous, minor adjustments required for stability and movement [12].

The modulation of postural tone relies on the excitability of motoneurons and interneurons, influenced by reticulospinal and vestibulospinal outputs, as well as contextual factors such as gravity exposure [13,14]. This dynamic process highlights the importance of evaluating tone not only through its mechanical properties but also through its functional contributions to postural stability and movement demands [4].

Concerning postural stability during daily tasks, the coordinated activation of agonist and antagonist muscles is required. For example, antagonist coactivation (CoA) plays a critical role in modulating joint stiffness and maintaining dynamic posture under varying biomechanical and environmental conditions [1,15]. A clear example of this regulation can be observed in the muscles of the ankle, which are essential for controlling the vertical projection of the centre of mass (COM) relative to the base of support in standing positions—the centre of pressure (COP) [16]. Furthermore, these muscles contribute significantly during transitions such as walking initiation [17] and sitting down [18,19]. Incorporating the study of CoA into the biomechanical quantification of postural tone seems crucial for a comprehensive understanding of postural control (PC) mechanisms [20,21]. Given its role in compensatory strategies, particularly in neurological conditions such as stroke, analysing CoA alongside stiffness parameters provides deeper insight into postural stability and its rehabilitation implications [22,23].

Postural stability indicators, such as COP displacement (dCOP), provide valuable insights into the resulting output of tonus modulation and reveal dysfunctions in neuromuscular coordination [24]. dCOP reflects the dynamic interplay between sensory input, motor output, and biomechanical constraints, making it a critical tool for assessing PC, particularly during tasks involving perturbations [25,26,27]. Impaired neuromuscular coordination is often manifested as a disruption in the balance between passive stiffness and active muscle coactivation, emphasising the importance of a complementary assessment that integrates active and passive components to better understand their contribution to PC in specific clinical populations, such as stroke survivors [28,29,30].

Recent advances in biomechanics and neuroscience suggest that PC and stability rely on neuromuscular adjustments made in discrete time intervals rather than continuously [31]. Studies using inverted pendulum models of postural sway have shown that long-term correlations and heterogeneity in time-series data arise from intermittent corrective actions rather than a continuous control strategy [31,32]. This paradigm provides a more nuanced understanding of postural stability regulation, demonstrating that non-linear methods may offer valuable insights beyond traditional stiffness-based assessments [31,33]. Specifically, evidence suggests that these control mechanisms contribute to postural instability in elderly populations and patients with Parkinson’s disease, further reinforcing the importance of integrating non-linear methodologies in neuromuscular control research [33,34]. This approach emphasises the need for alternative analytical models beyond linear assessments [35]. This progressive evolution of these models highlights the necessity of incorporating non-linear analytical approaches to better capture the complexity of muscle tone regulation [34,36].

To the best of our knowledge, no studies have specifically applied non-linear methodologies to the assessment of muscle tone. This represents a significant gap in the literature, as these tools are uniquely suited to quantify the temporal dynamics and complex interdependencies of neuromuscular control, supporting the application of entropy measures and Lyapunov (LyE) analysis to assess postural dynamics. These methods provide a deeper understanding of the inherent variability, adaptability, and stability of neuromuscular systems in varying conditions [31,34,37,38]. For instance, entropy measures can reveal the unpredictability and complexity of neural and muscular signals, while Lyapunov analysis assesses the stability of these systems by examining how small perturbations evolve over time [32,35,39,40]. These methods are particularly relevant in CNS impairments, such as in stroke survivors [32]. By addressing this gap, the incorporation of non-linear analyses into muscle tone assessment has the potential to advance our understanding of its regulation and contribute to the development of more precise, individualised therapeutic strategies [40].

Given these challenges, there is a growing need for improved assessment tools in the context of stroke rehabilitation, where dysfunctions in muscle tone modulation significantly affect motor recovery and functional outcomes. By complementing traditional methods with advanced biomechanical and electrophysiological approaches, this study seeks to enhance our understanding of muscle tone and its functional implications. Building on this background, this study aims to analyse the relationship between traditional stiffness quantification and muscle coactivation in both stroke and healthy individuals, using linear and non-linear measures of muscle coactivation and COP across functional tasks. To achieve this, we adopted a structured analytical approach comprising three key steps. First, we characterised intrinsic stiffness, functional stiffness, and antagonist muscle CoA in both groups. Second, we explored the relationship between intrinsic and functional stiffness and their association with antagonist CoA across the three functional tasks (standing, stand-to-sit, and gait initiation), while also examining CoA patterns between groups to identify potential neuromuscular adaptations in stroke survivors. Third, based on the hypothesis that CoA better reflects the neurophysiological mechanisms underlying postural tone, we conducted an in-depth analysis of CoA and centre of pressure (COP) dynamics using both linear and non-linear approaches. This multi-level analysis enabled us to establish connections between stiffness, muscle CoA, and postural stability, offering a comprehensive neurobiomechanical characterisation of postural tone in stroke survivors.

## 2. Materials and Methods

### 2.1. Study Design

A cross-sectional observational study was conducted to explore kinetic and electromyographic variables in post-stroke subjects (referred to as the “stroke group”), and subjects without a history of stroke and without self-reported disabilities (referred to as the “healthy group”), used as a reference for typical movement performance.

### 2.2. Participants

Twelve subjects who had a unilateral stroke episode at least six months earlier (5 women, 7 men) and 12 healthy subjects (8 women, 4 men) participated in this study. 

For the post-stroke subjects, the mean time between stroke and inclusion in this study was 26.7 months (SD = 12.10 months). All post-stroke subjects had experienced an ischemic stroke: 9 had an infarction in their left hemisphere, whereas 3 had an infarction in their right hemisphere, resulting in motor control dysfunction of the contralesional lower limb (CONTRA). To be included, participants were required to meet the following criteria:

(a) Have experienced a first-ever ischemic stroke, in chronic phase [41,42,43], involving the middle cerebral artery territory—specifically at the internal capsule—as revealed by computed tomography [42,43];
(b) Have a Fugl–Meyer Assessment of Sensorimotor Recovery After Stroke score below 34 in the lower limb subsection [43];
(c) Not have a grade 3 score for the Achilles’ tendon reflex;
(d) Present clinical signs of increased muscle tone (with a minimum score of 1 in the Modified Ashworth scale) in the calf muscles [44];
(e) Have the capacity to perform stand-to-sit (StandTS), maintain a stand position, and initiate gait without the use of orthoses;
(f) have provided written or verbal informed consent to participate in the study.

None of the subjects received antispastic medication during the study. Subjects were excluded if they met any of the following criteria:

(a) Had any cognitive deficits that could hinder communication and cooperation, assessed by the Mini-Mental State Examination [45];
(b) Had history of orthopaedic or neurological disorders known to affect stiffness, or other conditions (e.g., sensory impairment, diabetes, thrombophlebitis, history of lower limb surgery, or any orthopaedic or rheumatoid conditions) that could interfere with StandTS, stand position or gait;
(c) Were taking medication that could affect motor performance.

Data from the stroke group were compared with data obtained from the 12 subjects in the healthy group. All healthy subjects were sedentary adults without self-reported disabilities, recruited by direct invitation. They were excluded if they had met the following criteria:

(a) Altered mental state with interference in communication and cooperation [41,42,43];
(b) History or sign of neurological dysfunction [46];
(c) Presence of pain that could interfere with the performance of sitting, standing, or walking [43];
(d) History of anatomical deformities, osteoarticular, musculotendinous injury, or lower limb surgery in the last 6 months [43,46];
(e) Exposure to medication with interference with the motor performance of the lower limbs [41,42];
(f) Practice of moderate (i.e., at least 30 min, 5 days a week) or vigorous (i.e., at least 20 min, 3 days a week) levels of physical activity [47].

The study was approved by the Institutional Ethics Committee of the School of Health, Polytechnic of Porto (CE 1484). All participants provided their written informed consent to the experimental procedures, following the principles outlined in the Declaration of Helsinki.

### 2.3. Instruments

#### 2.3.1. Sample Selection and Characterisation

Inclusion and exclusion criteria were verified through a questionnaire to characterise the participants in terms of age, sex, weight, height, dominance (for both healthy and stroke groups), and time since stroke (for the stroke group).

Weight (Kg) and height (m) were assessed using a seca^®^ 760 scale (seca—Medical Scales and Measuring Systems^®^, Birmingham, UK), with a scale of 0.1 Kg; and a seca^®^ 222 stadiometer (seca—Medical Scales and Measuring Systems^®^, UK), with a 1 mm scale.

The physical activity level of healthy participants was assessed using the Brief Physical Activity Assessment Tool [48]. This questionnaire classifies participants as sufficiently or insufficiently active, showing good construct validity (0.40 ≤ k ≤ 0.64), sensitivity (0.75, 95% CI: 0.70–0.79), and specificity (0.74, 95% CI: 0.71–0.77) when compared to accelerometry and other physical activity questionnaires [49].

The Mini-Mental State Examination scale was used to assess cognitive level [50]. This scale evaluates five cognitive domains: memory, attention, calculation, language, and praxis, with a maximum score of 30 points. It has been adapted and validated for the Portuguese population, demonstrating a sensitivity of 63–73.4% and a specificity of 90–96.8% [51,52].

The Ashworth Scale was used to determine muscle tension during passive stretching. It is widely recognised for its intra- and inter-observer reliability [53].

The Portuguese version of the Fugl–Meyer Assessment of Sensorimotor Recovery After Stroke was applied to assess post-stroke sensorimotor impairment of the lower limb [54,55]. This version presents excellent internal consistency (α Cronbach = 0.99) and considers a score below 34 as indicative of sensorimotor impairment [56,57].

#### 2.3.2. Kinetic Data

The anteroposterior (Fx) and vertical (Fz) components of the ground reaction forces (GRF), force moments (Mx and Mz), and dCOP were assessed using a force plate, model FP4060-10 from Bertec Corporation (Columbus, OH, USA), connected to a Bertec AM 6300 amplifier.

To calculate ankle intrinsic stiffness (iStiff), angular position (°) and velocity (°/s), a Biodex System 4 Pro^®^ isokinetic dynamometer (Biodex Medical Systems, Inc., Shirley, NY, USA) was used. This dynamometer is a valid and reliable instrument (ICC = 0.97) [58]. A universal goniometer was used to ensure the neutral position of the ankle (0 ± 1.00°) on the lever axis of the isokinetic dynamometer.

#### 2.3.3. EMG Data

To guarantee that there was no muscle activity during ankle iStiff, surface electromyography (EMG) was assessed using a Biopac MP150 workstation that was synchronised with the isokinetic dynamometer (Biopac Systems, Inc., Goleta, CA, USA). Active TSD150B electrodes with a bipolar configuration were positioned on the tibialis anterior (TA), soleus (SOL), and gastrocnemius medialis (GM) muscles.

During the assessment of functional tasks, bilateral EMG activity of TA, SOL, and GM muscles was collected to assess antagonist coactivation (CoA), using a wireless EMG acquisition system (TrignoTM, Delsys Inc., Natick, MA, USA). Pre-amplified bipolar differential electrodes (Trigno Avanti Sensor model), with a rectangular configuration of two AgCl bars placed in parallel (inter-electrode distance of 10 mm), were used to collect the EMG signal. EMGworks software 4.0 (Delsys Inc., USA) was used to analyse the EMG signal quality. An Electrode Impedance CheckerVR (Noraxon, Scottsdale, AZ, USA) was also used to measure the level of skin impedance. The data from this system and kinetic data were collected synchronously using the Qualisys Track Manager (Qualisys AB^®^, Gothenburg, Sweden).

### 2.4. Procedures

Data were collected at the Centre for Rehabilitation Research (CIR), School of Health, Polytechnic Institute of Porto, in a controlled environment. Each task was performed by a single researcher to minimise inter-rater variability. Prior to data collection, anthropometric measures were recorded for each participant.

#### 2.4.1. Skin Preparation and Electrodes Placement

The skin surface over the belly of the selected lower limb muscles was prepared by shaving and removing dead skin cells and non-conductive elements with isopropyl alcohol (70%) and an abrasive pad to reduce its electrical resistance. An electrode impedance checker was used to ensure that impedance levels were lower than 5 kΩ [59].

Electrodes were placed according to the Surface ElectroMyoGraphy for the Non-Invasive Assessment of Muscles (SENIAM) [59] and the anatomical references from [60] (Table 1). Electrode placement was confirmed by palpation.

#### 2.4.2. Data Acquisition

- Ankle intrinsic stiffness

Participants were seated on the Biodex system adjustable chair with the knee in full extension (0°) and the hip at 80° flexion and 0° DF. This orientation of the lower limb was chosen according to the Biodex System 4 Pro^®^ dynamometer user manual (Biodex Pro Manual, Applications/Operations; Biodex Medical Systems, Inc., Shirley, NY, USA) to obtain ankle iStiff values in a position as similar as possible to the uSt position used during the functional tasks under study (0° knee flexion and 0° ankle DF)—StandTS, uSt, and gait initiation (GI) [61]. Adjustable straps provided stabilisation at the chest, waist, and thigh, and the foot was secured using two adjustable straps. The anteroposterior (AP) ankle centre of rotation was aligned with the rotational axis of the dynamometer. Three passive DF were imposed on both limbs of post-stroke and healthy subjects, at a velocity of 5°/s, from maximum comfortable plantarflexion (PF) to maximum comfortable DF. A velocity of 5°/s is considered slow enough to be used as a subthreshold for evoking a stretch reflex [62] and has been used in previous studies [42]. Pauses of 1 s separated each passive DF, during which the ankle was placed at a neutral position. During testing, subjects were instructed to relax all muscles in the lower limb and to refrain from interfering with the passive movements. Before data collection, each subject had familiarisation trials. In addition, these testing procedures decrease thixotropy [63] and the stress relaxation phenomena [64].

Data concerning the angular position and torque from the dynamometer were collected at 1000 Hz, along with EMG. Additionally, to ensure that reflexive or voluntary muscle activity was not elicited during the passive movements, EMG signals from the SOL, GM, and TA muscles from the stroke group in the CONTRA and ipsilesional (IPSI) lower limbs, and in the healthy group in the dominant (DOM) and non-dominant (NDOM) lower limbs, were acquired at the same sample rate using the previously described surface EMG system (Biopac Systems, Inc., Goleta, CA, USA).

- Functional stiffness

Participants maintained the uSt position with minimal movement, with feet placed naturally on the force plate, configured at a 1000 Hz sampling frequency [65], and the upper limbs parallel to the trunk (according to individual capacity), with a visual reference at eye level placed 2 m away, for 30 s [66].

- Antagonist coactivation in Standing, StandTS, and GI

For the functional tasks under study, data were collected using the force plate and bilateral EMG from the SOL, GM, and TA muscles [18,41,60,65]. The necessary repetitions were performed to obtain three valid ones [67,68], and a minimum interval of one minute between repetitions was allowed to avoid fatigue [19].

Subjects assumed the uSt position with minimal movement [69], with their feet spontaneously placed on the force plate [17,70], their upper limbs parallel to the trunk (according to individual capacity), and a visual reference at eye level placed 2 m away [66]. This initial (starting) uSt position was marked on the top of the force plate to ensure consistency across trials.

In order to collect data in uSt, participants maintained this position for 30 s [66,71]. Both groups were assessed: healthy (DOM and NDOM) and stroke (CONTRA and IPSI).

In StandTS data acquisition, subjects, starting from the uSt position, were instructed to perform the task at their normal speed using the above-mentioned visual reference [72], without resorting to the upper limbs or moving their feet after the verbal command “Please, sit”, thus ensuring the validity of the repetition [73,74,75]. Both groups were assessed: healthy (DOM and NDOM) and stroke (CONTRA and IPSI).

In GI data collection, subjects, in the starting uST position, were asked to start walking at their usual speed over a predetermined distance of 5 m [65], upon hearing the voice command “Please, walk”. Repetitions were considered valid when performed with at least three steps [76,77,78,79]. No additional instructions were given, so as not to influence the accomplishment of the task [80,81]. Both groups were assessed: swing (SWING) and stance (STANCE) lower limbs from healthy and stroke participants.

If participants displayed hesitation due to unclear understanding of the instructions or required physical assistance, the trial was conducted again [82]. Before data collection, participants were allowed to perform practice trials to warm up and become familiar with the experimental setup and procedures [43,72]. All participants wore standardised footwear, consisting of flat shoes with rubber soles and laces, properly fitted to their size [60,83]. No orthotics or assistive devices were used by participants during the data collection process [60].

**Figure 1:**
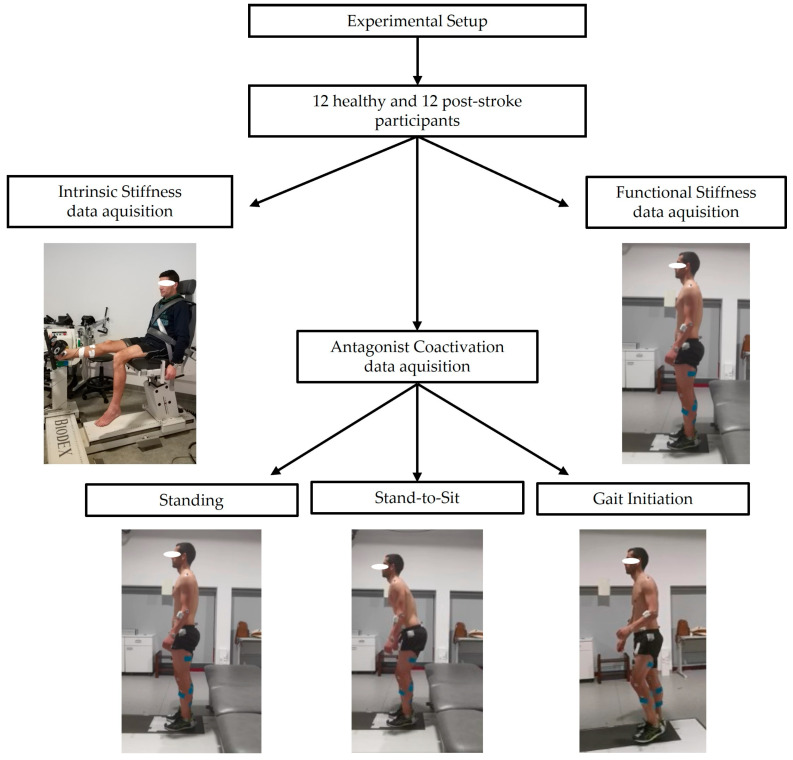
Experimental setup for data acquisition on both healthy and stroke groups: “ankle intrinsic stiffness”, “functional stiffness”, and “antagonist coactivation” in standing, stand-to-sit, and gait initiation.

A representative diagram of the data acquisition process is represented below (Figure 1). Both the healthy and stroke groups were evaluated using the same experimental protocols.

#### 2.4.3. Data Processing

- Ankle intrinsic stiffness

The software Acqknowledge^®^ version 3.9 was used to process and analyse data from the MP150 system (Biopac Systems Inc., Goleta, CA, USA), namely torque, angular position, and EMG. EMG signals were band-pass filtered with a 4th-order zero-phase shift Butterworth at 20–500 Hz and root-mean-squared using a 100-sample moving window [84,85].

Torque and angular position data corresponding to the bilateral DF in the healthy and stroke groups were the only part of the cycle considered [61]. To eliminate the confounding effects of inertia and to ensure the analysis of constant velocity data, the first and last degrees of DF were not included in the analysis [86,87]. Repetitions with the presence of voluntary and/or reflex muscle activity were not considered valid [88]. Trials in which EMG activity in the SOL, GM, or TA exceeded 5 µV were excluded, considering that the normal amplitude of EMG signals in resting muscles is around 2–5 µV [89].

**Figure 2:**
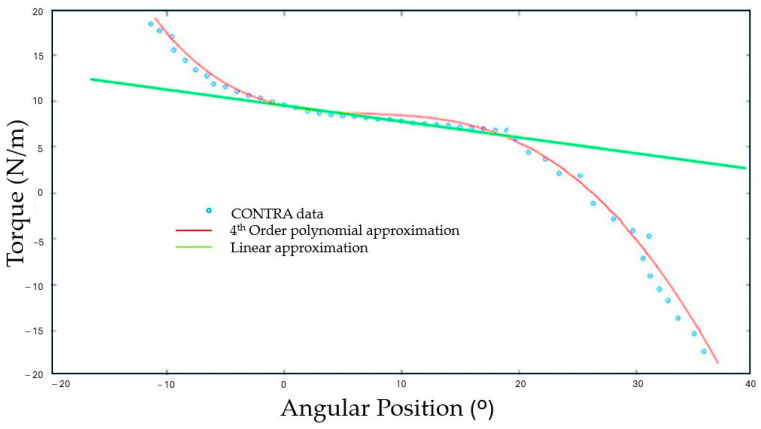
Torque (Nm) versus angular position (°) with polynomial and linear fitted curves.

Ankle iStiff (Nm/°) was quantified for each limb (DOM, NDOM, CONTRA, and IPSI) from the three valid repetitions by constructing a scatter plot in Matlab™ R2021a software (The MathWorks^®^, Inc., Natick, MA, USA) using 4th-order polynomial equations relating angular position and torque. As an example, Figure 2 demonstrates a scatter plot generated from the CONTRA limb of a stroke participant, confirming the appropriateness of this model for capturing the observed data (R^2^ = 0.9989).

*F*(*x*) is the torque corrected for gravity, *x* is the angular position, and a–e are constants (1) [88,90]:

*F*(*x*) = *ax*4 + *bx*3 + *cx*2 + *dx* + *e* (1)

For each angular position, stiffness values were calculated using the first derivative *F*’(*x*)—the slope of Equation (2):

*dy/dx* = 4*ax*3 + 3*bx*2 + 2*cx* + *d* (2)

where *dy*/*dx* represents the intrinsic stiffness at each angular position [88,91].

The coefficient of determination (r2) values were obtained by calculating the two equations above (1) and (2). The arithmetic mean of the stiffness values obtained from the three valid repetitions was calculated [88,92]. The iStiff values were analysed at the ankle position closest to the standing position [61]. In order to present a value that is representative of the overall behaviour, the average of the iStiff values obtained across the individual’s repetitions was also calculated.

- Functional stiffness

Force plate data, namely COP data, were low-pass filtered with a 4th-order zero-phase shift Butterworth filter at 20 Hz [70].

Given the importance of the plantar and dorsiflexor muscles in controlling the vertical projection of the centre of mass (COM) -the centre of gravity- in the anteroposterior (AP) direction in the uSt position [93], the main focus was on stiffness in this direction. In order to process and analyse the functional stiffness (fStiff) values, a routine produced in Matlab™ R2021a software (The MathWorks^®^, Inc., Natick, MA, USA) was used, based on a mathematical model proposed by [94,95], in which the direct estimation of stiffness results from the real-time measurement of the COM and COP positions (pCOP).

The COP displacement (dCOP) value was recorded during a stable time interval in the standing position (middle 15 s), with the feet positioned on the force plate.

For each of the three valid repetitions, a linear regression line was drawn between the angle of oscillation—formed between the vertical line connecting the ankle to the COM and the line of gravity, and the moment of force of the dCOP and the line of gravity—and the moment of the ankle, representative of the two lower limbs. The calculation of the fStiff for each repetition was then performed, after which the average of the three valid repetitions was calculated for both healthy and stroke groups.

**Figure 3:**
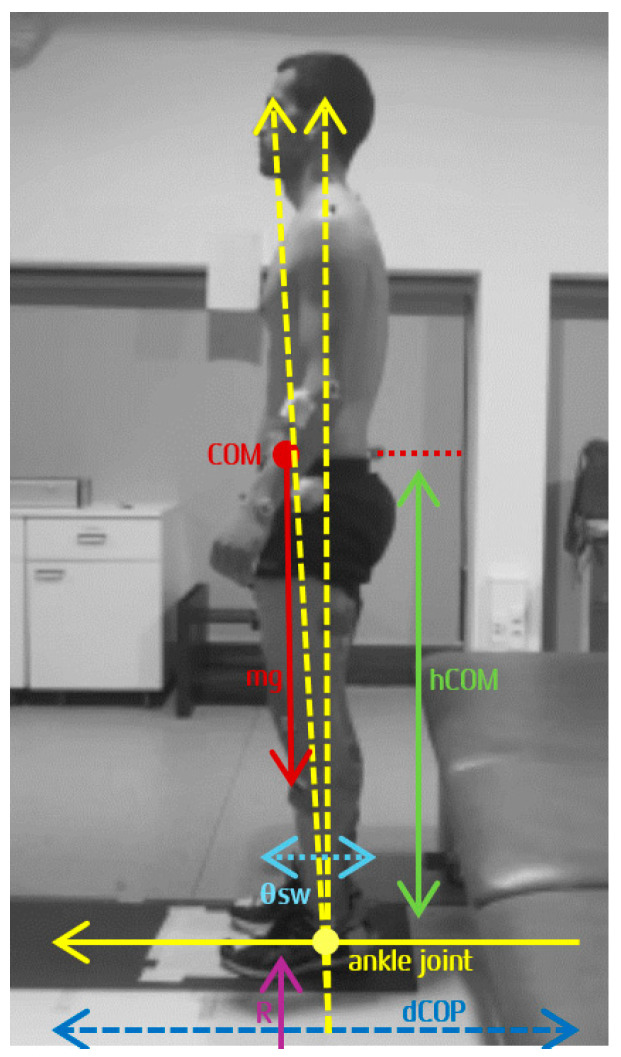
Adapted from Winter et al. (2001) [95]: Inverted pendulum model showing the variables: centre of mass (COM), centre of pressure (COP), body weight (mg), height of COM (hCOM), vertical component of ground reaction force (R), anteroposterior sway (θsw), and COP displacement (dCOP), from which direct measure of muscle stiffness can be estimated.

According to Winter et al. (1998), AP stiffness in uSt can be calculated from the readily measured time records of COP and COM [94]. Figure 3 presents the commonly used inverted pendulum model in the sagittal plane [95].

The COM and the COP are measured relative to the ankle joint, with the COM located at a distance (hCOM) above the ankle joint. The sway (θsw) is defined by the angle of the line joining the ankle to the COM. Body weight (mg) is the force of the body above the ankle joint, and the vertical reaction force (R)—which does not include the reaction force of the feet, considered essentially stable during quiet standing—is equal to body weight. The sum of the left and right ankle moments, *AM*, is given by the following Equation (3):

*AM* = *R* × *COP* = *Kg* × *COP* (3)

*θsw* = *COM/h*

*Stiffness Ka* = *dAM/dθsw*

- Antagonist coactivation and COP in standing, StandTS and GI

Antagonist coactivation (CoA) in standing (DOM and NDOM lower limbs in healthy; CONTRA and IPSI lower limbs in stroke), StandTS (DOM and NDOM lower limbs in healthy; CONTRA and IPSI lower limbs in stroke), and GI (stance and swing lower limbs in both groups) was calculated according to the following steps. Registered EMG values from a 15 s window, with the subjects stable in the uSt position before performing StandTS and GI tasks (baseline), were used to normalise signals from those tasks. Data from dCOP at the beginning of each task were also analysed when the condition was satisfied: both feet on only one force plate. The identification of the postural phase initial (M0) and final (M1) moments was based on dCOP in the AP and ML directions [65,96,97]. For GI, T0 was identified by the first temporal moment where dCOP ML or AP showed displacement (first peak in dCOP ML signal) [98]. M0 was then defined as the beginning of the time interval ≥ 50 ms, calculated within a temporal window of −450 to +50 ms in relation to T0, where dCOP in the ML or AP direction was exceeded the mean of the baseline ± 3 standard deviations (Baseline ± 3 STD). M1 was defined as the time instant at which the same signal’s curve inverted [70]. For the postural phase of StandTS, the same criteria were applied. During the postural phase of these three functional tasks, the root-mean-square of EMG activity was calculated between the M0-M1 interval. For all tasks, EMG and COP data were time-normalised to a 1 s window. To calculate CoA, the following formula was used (4) [99]:

(4) $$CoA(\%)=\frac{antagonist\;activity}{agonist+antagonist\;activity}\times 100$$

For CoA calculation, the SOL and GM muscles during standing [16,63], and the TA muscle during the other tasks [70,100,101], were considered agonists. These variables were calculated according to the formulae presented in Table 2 [21,99,102,103].

Linear CoA variables (expressed in %) were characterised by measures of magnitude and variation. To perform the non-linear analysis, the largest Lyapunov exponent was employed to evaluate system stability by examining divergence or convergence over time [36]. Additionally, multiscale entropy (MSE) was calculated to quantify system complexity, capturing adaptability and variability in COP-AP and coactivation variables. Both metrics were computed using custom scripts developed in Matlab™ R2021a (The MathWorks^®^, Inc., Natick, MA, USA).

Multiscale entropy (MSE) is a well-established approach for evaluating the complexity of time series data across multiple temporal scales, enabling the identification of intricate dynamics in physiological signals [38,104]. The sample entropy (SE) algorithm was used to quantify regularity at each scale, building on the concept of approximate entropy, to provide an assessment of complexity [105,106].

The complexity index (CI) was used as the primary dependent variable to investigate differences between the stroke and healthy groups in terms of MSE. The analysis adhered to the protocols outlined by Costa et al. [107] and Goldberger et al. [108]. Initially, the SE was calculated according to the following Equation (5) [109]:

(5) $$SE\left(m,\;r,\;N\right)=-In\frac{Um+1\left(r\right)}{Um\;\left(r\right)}$$

where *S*E is the sample entropy value, m is the number of samples in comparison (m = 2), r is the similarity threshold (0.2 of the standard deviation signal), N is the number of samples (1000 points), and U is the probability of the samples falling within r.

The original time series was subjected to a coarse-graining process (6), enabling the evaluation of complexity across three time scales:

(6) $$y_{j}^{\tau m}=\left(\frac{1}{\tau m}\right)\sum_{i=\left(j-1\right)\tau m+1}^{j\tau }x_{1},1\leq j\leq \frac{N}{\tau m}$$

where $y_{j}^{\tau m}$ represents the new time series, generated by averaging non-overlapping data points from the original time series according to the scale factor (*τm*). This coarse-graining procedure facilitates the computation of SE across all time scales, ranging from 1 to *τm*.

In Equation (7), the CI is determined through the numerical integration of individual SE values across all evaluated time scales:

(7) $$C_{1}=\sum_{j=1}^{N}S_{E}(i)$$

where *S*_E_ is the SE value calculated at each individual time scale (*i*).

The LyE algorithm, originally introduced by Wolf et al. in 1985,, is represented by a single equation. In this expression (8), L denotes the distance between points, t indicates the time lag, and *M* represents the total number of replacement steps [110,111].

(8) $$\lambda \left(i\right)=\frac{1}{M}\;\sum_{{K=K}_{min}}^{Kmax}\left(\frac{1}{K.t}\;ln\frac{L_{i+K}}{L_{i}}\;\right)$$

LyE calculation involved reconstructing the phase space following the approach outlined by Broomhead and King [112,113]. This reconstruction was achieved by creating multiple delayed copies of the time series, using a time lag (τ) to facilitate state space representation for estimating non-linear parameters. In this study, a time lag of τ = 1 and an embedding dimension of m = 2 were selected, based on previous methodological recommendations and the relatively simple dynamics of the system under analysis [114]. The algorithm quantifies the divergence or convergence of trajectories within the reconstructed phase space, providing a numerical measure of the system’s sensitivity to initial conditions [115]. Positive values indicate divergence of trajectories, reflecting greater instability and chaotic dynamics [116]. Conversely, negative values exhibit convergent trajectories, suggesting more robust postural control [117]. This measure highlights the adaptive complexity of the PC system, balancing between stability and chaos in response to varying task demands [40].

#### 2.4.4. Statistical Analysis

Data analysis was conducted using IBM SPSS Statistics^®^ version 30.0 (IBM Corporation, Armonk, NY, USA), with a significance threshold set at 0.05. The Shapiro–Wilk test was employed to evaluate the normality of continuous variables. Descriptive statistics were used to summarise the data, reporting the median and 25th and 75th percentiles for quantitative variables, since the normality assumption was not satisfied [118]. Non-parametric methods were applied to assess group comparisons (Mann–Whitney test) and correlations between variables (Spearman’s Correlation). The strength of correlations was classified according to the British Medical Journal guidelines: very weak for correlation coefficients of 0–0.19, weak for 0.2–0.39, moderate for 0.4–0.59, strong for 0.6–0.79, and very strong for 0.8–1.0 [100].

## 3. Results

Participants’ characteristics are summarised in Table 3. No significant differences were found between the healthy and stroke groups; therefore, both groups were considered comparable.

Descriptive analysis was conducted to characterise iStiff, fStiff, and CoA during standing, StandTS, and GI in both stroke and healthy groups (Table 4).

### 3.1. Intrinsic Stiffness, Functional Stiffness and Antagonist Coactivation Correlations

#### 3.1.1. Intrinsic Stiffness and Functional Stiffness

Table 5 presents the results of the correlation analysis between iStiff and fStiff variables. Specifically, iStiff values from the DOM and NDOM lower limbs of the healthy group were correlated with the functional stiffness values of the same group (A). Similarly, iStiff values from the IPSI and CONTRA lower limbs of the stroke group were correlated with the fStiff values of the stroke group (B). Additionally, intergroup correlations were performed within iStiff. DOM of the healthy group was correlated with IPSI of the stroke group, and NDOM of the healthy group was correlated with CONTRA of the stroke group (C). Finally, fStiff values were correlated between the stroke and healthy groups (D) to explore potential overarching patterns in stiffness behaviour across groups.

The correlation between iStiff IPSI and fStiff STROKE was moderate (*r* = 0.623) and statistically significant (*p* = 0.030). All other correlations were weak to moderate and not statistically significant (*p* > 0.05).

#### 3.1.2. Intrinsic Stiffness and Antagonist Coactivation

Table 6 presents the correlation coefficients between iStiff, and the CoA values of different muscle pairs assessed during three functional tasks: standing, StandTS, and GI, in both stroke and healthy groups. This analysis aimed to explore the relationships between intrinsic mechanical properties and neuromuscular activation patterns under varying functional demands.

In the healthy group, for iStiff DOM during the standing task, a significant positive correlation was observed with TA/SOL (*r* = 0.448, *p* = 0.048). No other significant correlations were found for other muscle pairs or tasks.

For the stroke group, iStiff CONTRA during the StandTS task, a significant positive correlation was observed with Do/Ve (*r* = 0.403, *p* = 0.048). No significant correlations were found for other tasks or muscle pairs.

#### 3.1.3. Functional Stiffness and Antagonist Coactivation in Standing, Stand-to-Sit and Gait Initiation

The following table (Table 7) presents the Spearman’s correlation coefficients between fStiff and CoA values of different muscle pairs, evaluated during three functional tasks: standing, StandTS and GI.

In the healthy group, correlations in standing showed weak negative relationships for most muscle pairs, with no significant associations (*p* > 0.05). In StandTS, correlations remained weak, negative, and non-significant across all evaluated pairs. For GI, correlations were also weak and non-significant.

In the stroke group, during standing, correlations were mostly weak and positive for CONTRA muscle pairs, with no significant associations (*p* > 0.05). In StandTS, IPSI muscle pairs exhibited weak negative correlations, all of which were non-significant. For GI, moderate positive correlations were observed for the GM/TA STANCE muscle pair (*r* = 0.587, *p* = 0.045), suggesting a significant association.

#### 3.1.4. Antagonist Coactivation in Standing, Stand-to-Sit and Gait Initiation in Healthy (Dominant vs. Non-Dominant Side) and in Stroke (CONTRA vs. IPSI Lesional Side)

Table 8 presents the Spearman’s correlation coefficients (r) and corresponding *p*-values analysing the relationships between CoA of the DOM and NDOM lower limbs in the healthy group, and between the IPSI and CONTRA lower limbs in the stroke group. The analysis evaluated these correlations across three functional tasks: standing, StandTS and GI.

In the healthy group (DOM vs. NDOM), the standing task showed moderate positive correlations for most muscle pairs, with significant associations for TA/SOL (*r* = 0.832; *p* = 0.001) and TA/GM (*r* = 0.804; *p* = 0.002). For StandTS, significant correlations were found for GM/TA (*r* = 0.762; *p* = 0.004).

In the stroke group (IPSI vs. CONTRA), during standing, strong correlations were observed for SOL/TA (*r* = 0.776; *p* = 0.003) and GM/TA (*r* = 0.699; *p* = 0.011).

### 3.2. COP Related Variables

**Figure 4:**
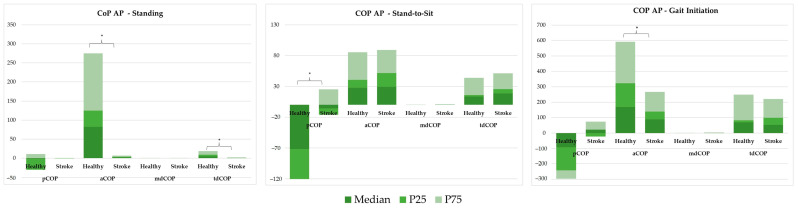
Linear analysis of centre of pressure anteroposterior behaviour, comparing COP position (pCOP), amplitude (aCOP), mean displacement (mdCOP), and total displacement (tdCOP) between healthy individuals and stroke patients across three tasks: standing, stand-to-sit (STSit), and gait initiation (GI). * represents significant differences.

**Figure 5:**
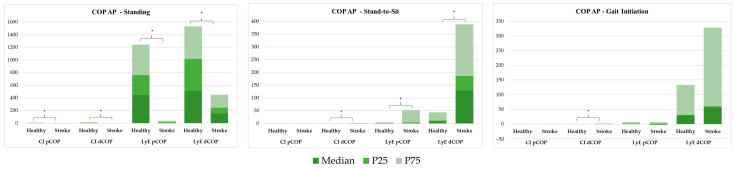
Non-linear analysis of centre of pressure (COP) anteroposterior (AP) behaviour, using the complexity index (CI) and Lyapunov exponent (LyE), to compare COP position (pCOP) and total displacement (dCOP) between healthy individuals and stroke patients across three tasks: standing, stand-to-sit (STSit), and gait initiation (GI). * represents significant differences.

The CoP AP behaviour in standing, StandTS and GI functional tasks was analysed using both linear (Figure 4) and non-linear (Figure 5) approaches.

#### 3.2.1. Linear Analysis

Figure 4 shows a linear analysis comparing COP position (pCOP), amplitude (aCOP), mean displacement (mdCOP), and total displacement (tdCOP) between healthy and stroke groups across three tasks: standing, StandTS, and GI.

In the standing position, aCOP and tdCOP were significantly reduced in the stroke group (aCOP = 2.159 vs. 82.617; tdCOP = 0.670 vs. 5.238; *p* < 0.001). In the stroke group, there were significant differences in pCOP during the stand-to-sit task (−5.380 vs. −71.715, *p* < 0.001), and significantly reduced aCOP (88.553 vs. 169.611, *p* = 0.005) and increased pCOP (21.648 vs. −91.580, *p* = 0.001) in the GI task.

#### 3.2.2. Non-Linear Analysis

Figure 5 demonstrates non-linear metrics comparison, including the CI and LyE, between healthy and stroke groups across three tasks: standing, StandTS and GI.

In the standing position, the stroke group exhibited significantly lower CI for pCOP (0.282 vs. 1.925, *p* < 0.001) and dCOP (0.371 vs. 2.468, *p* < 0.001), as well as significantly lower LyE for pCOP and dCOP (dCOP = 152.976 vs. 512.634, *p* < 0.001). In StandTS, the stroke group showed reduced CI dCOP (0.269 vs. 0.142, *p* = 0.003) and LyE dCOP (128.959 vs. 7.391, *p* < 0.001), and LyE pCOP 3.466 vs. 1.091, *p* < 0.001). In the GI task, the stroke group showed reduced CI for dCOP (0.393 vs. 0.115, *p* = 0.038).

### 3.3. Antagonist Coactivation Analysis

**Figure 6:**
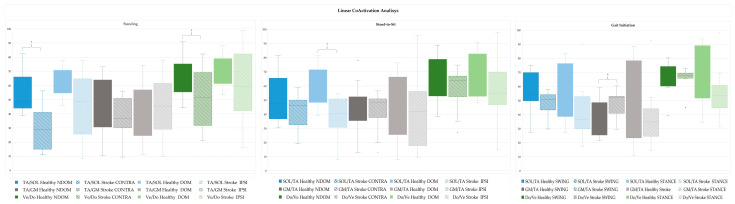
Linear analysis of antagonist coactivation between muscle pairs in the dominant and non-dominant lower limbs during standing and stand-to-sit, and swing and stance lower limbs during gait initiation in the healthy group; and between the ipsi and contralesional side during standing and stand-to-sit, and swing and stance lower limbs during gait initiation in the stroke group. * *p*-value < 0.05: Mann–Whitney Test.

**Figure 7:**
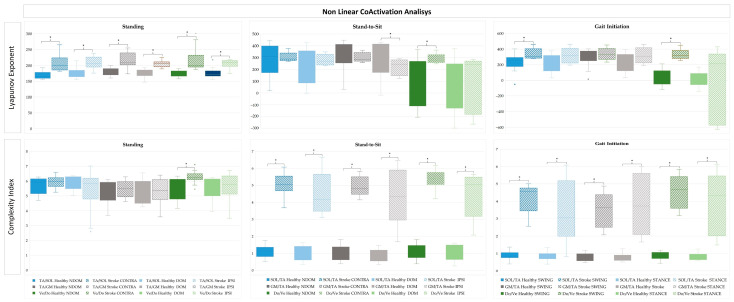
Non-linear analysis of antagonist coactivation between muscle pairs, using the LyE and CI, in the dominant and non-dominant lower limbs during standing and stand-to-sit, and swing and stance lower limbs during gait initiation in the healthy group; and between the ipsi and contralesional side during standing and stand-to-sit, and swing and stance lower limbs during gait initiation in the stroke group. * *p*-value < 0.05: Mann–Whitney Test.

The CoA in standing, StandTS and GI functional tasks was analysed using both linear (Figure 6) and non-linear (Figure 7) approaches.

#### 3.3.1. Linear Antagonist Coactivation in Standing, Stand-to-Sit and Gait Initiation in Healthy and Stroke

Figure 6 presents a linear analysis comparing CoA between lower limb muscle pairs of the DOM and NDOM during standing and StandTS, and between swing and stance during GI in the healthy group; and between the IPSI and CONTRA during standing and StandTS, and between swing and stance during GI in the stroke group.

In the linear EMG analysis of standing, significant reductions in SO/TA and Do/Ve CoA were observed on the CONTRA side in the stroke group compared to the healthy group (28.776 vs. 55.489, *p* < 0.001; 51.804 vs. 69.975, *p* = 0.021, respectively). In the StandTS task, the stroke group exhibited significantly lower SO/TA CoA on the CONTRA side compared to the healthy group (46.424 vs. 60.904, *p* = 0.007). For GI, significant differences were observed in GM/TA CoA during swing, with the stroke group showing higher CoA compared to the healthy group (51.926 vs. 39.741, *p* = 0.038).

#### 3.3.2. Non-Linear Antagonist Coactivation in Standing, Stand-to-Sit and Gait Initiation in Healthy and Stroke

A non-linear metrics comparison of CoA in lower limb muscle pairs was conducted using the LyE and CI (Figure 7) measures. In the healthy group, comparisons between the DOM and NDOM in standing and StandTS, and between swing and stance during GI, and between the IPSI and CONTRA during standing and StandTS, and between swing and stance during GI in the stroke group were made.

In the non-linear EMG analysis of standing, LyE for SOL/TA and Do/Ve CoA were significantly higher in the stroke group (*p* < 0.001), with CI for Do/Ve CoA also showing substantial differences (*p* < 0.001). In StandTS, the stroke group (CONTRA and IPSI sides) showed elevated CI for SOL/TA and Do/Ve CoA compared to healthy counterparts (*p* < 0.001). LyE for Do/Ve CoA was significantly higher on the stroke contra side (*p* = 0.011), whereas the DOM lower limb in the healthy group remained more stable. For GI, the stroke group exhibited significantly higher LyE for SOL/TA CoA in the swing (*p* = 0.090 *) and stance (*p* < 0.001) lower limbs. CI for the SOL/TA and CI GM/TA muscle pairs CoA were markedly elevated in the stroke group for both swing and stance lower limbs (*p* < 0.001). LyE for Do/Ve CoA showed a significant increase in the stroke swing limb (*p* < 0.001).

## 4. Discussion

This study aimed to analyse the relationship between traditional stiffness quantification and muscle CoA in stroke and healthy individuals, using both linear and non-linear measures of CoA and COP dynamics across functional tasks. The findings provide valuable insights into the neurobiomechanical underpinnings of muscle tone, highlighting significant alterations in the stroke group across all domains of analysis, including stiffness metrics, coactivation patterns, and postural control. These results underscore the complexity of muscle tone regulation and its implications for motor function, particularly in stroke survivors.

### 4.1. Intrinsic and Functional Stiffness Correlations

A key finding of this study was the significant positive correlation between CONTRA iStiff and fStiff in stroke participants. This relationship emphasises the interdependence between passive and functional components of muscle tone, consistent with previous findings that underscore the role of stiffness in compensating for neuromuscular impairments [3,4]. The compensatory role of the CONTRA limb in functional tasks highlights broader neuromuscular adaptations post-stroke, as documented by studies investigating contralesional contributions to motor performance [28,29].

In contrast, the absence of significant correlations involving IPSI iStiff suggests a more complex and variable relationship between iStiff and functional outcomes in this limb. Task-specific compensatory mechanisms and the heterogeneity of stroke severity may explain these findings, as previously observed in the variability of neuromechanical adaptations post-stroke [9,10]. The reliance on CONTRA stiffness suggests a potential target for therapeutic interventions aimed at modulating stiffness asymmetries to enhance overall motor function [3].

In the healthy group, the lack of significant correlations between iStiff and fStiff indicates that iStiff does not strongly reflect functional motor output under non-pathological conditions. This observation aligns with prior research demonstrating that healthy neuromuscular systems rely primarily on dynamic control strategies to optimise task execution, rather than on passive stiffness [4].

### 4.2. Intrinsic Stiffness and Antagonist Coactivation

The relationship between iStiff and CoA revealed task- and group-specific patterns. In the healthy group, a significant correlation between DOM iStiff and TA/SOL CoA during the standing task suggests a potential interaction between passive stiffness and muscle activation in stabilising static postures. These results align with the findings of Ivanenko and Gurfinkel (2018), who highlighted the importance of intrinsic muscle properties in maintaining postural stability [12].

In the stroke group, the positive correlation between CONTRA iStiff and Do/Ve CoA during the StandTS task indicates that CONTRA stiffness may contribute to enhancing stability during transitional movement, likely through compensatory mechanisms. This aligns with findings from previous studies, such as Lee et al. (2019), which associated exaggerated CoA patterns with efforts to counteract instability in dynamic tasks [29]. Nonetheless, the lack or inconsistency of correlations in other tasks and muscle pairs underscores the heterogeneity of neuromuscular adaptations post-stroke, as highlighted by Joshi et al. (2022) [8].

### 4.3. Functional Stiffness and Antagonist Coactivation

The weak correlations between fStiff and CoA in both the healthy and stroke groups suggest that fStiff does not consistently reflect CoA patterns. In the healthy group, the lack of significant associations across tasks reinforces the notion that fStiff is predominantly regulated by dynamic motor strategies rather than by intrinsic properties [1].

In the stroke group, a moderate positive correlation between fStiff and GM/TA CoA during the STANCE limb of GI highlights a compensatory strategy to mitigate reduced postural stability. This is in accordance with the work of Stergiou and Decker (2011), who observed that stroke survivors often rely on heightened CoA strategies to stabilise movements in dynamic tasks [32]. However, the absence of significant correlations across other tasks underscores the task-specific nature of these compensatory mechanisms, influenced by varying biomechanical demands [28].

### 4.4. Antagonist Coactivation

The analysis of interlimb CoA revealed distinct patterns in healthy and stroke participants. In the healthy group, strong correlations between DOM and NDOM CoA patterns during static tasks, such as standing, reflect the high degree of bilateral coordination and symmetry typical of non-pathological motor control [24].

In contrast, in the stroke group, significant correlations between IPSI and CONTRA CoA patterns during standing suggest that compensatory neuromuscular strategies enable interlimb coordination under static conditions. However, the absence of significant symmetry during dynamic tasks, such as GI, indicates that the increased coordination demands of more complex movements disrupt bilateral integration post-stroke. These findings are consistent with studies showing reduced bilateral motor integration in tasks requiring rapid or complex adjustments [17]. This observed asymmetry strengths the need for rehabilitation strategies aimed at improving bilateral coordination and minimising reliance on maladaptive compensatory mechanisms [119].

### 4.5. CoP Dynamics

The analysis of COP dynamics in this study revealed significant postural impairments in stroke participants compared to healthy individuals across functional tasks. By integrating linear and non-linear approaches, the findings underscore the multifaceted nature of postural control deficits in stroke, providing deeper insights into the neuromuscular dysfunctions underlying these impairments.

Stroke participants demonstrated significantly reduced aCOP and tdCOP during static standing and GI tasks. These reductions indicate a diminished range of movement and an impaired ability to generate appropriate postural adjustments—both crucial for maintaining stability. These deficits are consistent with previous research identifying limited weight-shifting capacity and long-term impairments as hallmarks of stroke-related motor dysfunction [24,120]. Additionally, the altered pCOP observed in stroke participants during GI suggests insufficient anticipatory postural adjustments (APAs), which are important for effective movement preparation. The presence of positive pCOP values in the stroke group likely reflects a compensatory strategy to respond to their reduced ability to efficiently shift weight in response to task-specific biomechanical demands. This finding corroborates studies reporting disrupted APAs and impaired motor planning in stroke survivors, which contribute to reduced functional capacity [4,70].

Non-linear metrics provided additional insights into the complexity and adaptability of postural control strategies. Stroke participants exhibited significantly lower CI values for both pCOP and dCOP during standing and StandTS transitions. This reduction in CI reflects a decline in postural complexity, emphasising the stroke group’s reliance on less flexible and more rigid control strategies. These findings align with previous studies demonstrating that reduced postural variability in stroke survivors indicates compromised neuromuscular adaptability [32,121].

Similarly, LyE values during standing and StandTS transition show impaired dynamic stability and a reduced capacity to recover from perturbations. As a reliable marker of stability, lower LyE values imply diminished robustness in postural control systems. These results are consistent with research indicating that stroke survivors exhibit reduced dynamic stability, particularly during tasks requiring continuous adjustments [35,36].

Interestingly, while CI measures were reduced during GI tasks, no significant differences in LyE were observed. This discrepancy may reflect the specific biomechanical demands of GI, which might involve fewer perturbation recoveries compared to transitional movements such as StandTS. This observation accentuates the importance of complementary metrics such as CI and LyE in capturing distinct dimensions of postural control [34].

### 4.6. Antagonist Coactivation Patterns

This study identified significant adaptations in CoA patterns in stroke participants compared to healthy participants, with marked differences across functional tasks. These findings provide important insights into the neuromuscular dysfunctions underlying postural and movement control impairments in stroke survivors. By integrating linear and non-linear analyses, the results offer a nuanced understanding of CoA dynamics and their implications for post-stroke rehabilitation. During the standing task, stroke participants exhibited significantly reduced CoA in the TA/SOL and Ve/Do muscle pairs on the CONTRA side compared to healthy individuals. These reductions reflect impaired postural control, as antagonist activity plays a crucial role in stabilising upright posture under static conditions. This diminished CoA aligns with previous findings that stroke survivors experience weakened neuromuscular coordination and reduced reciprocal inhibition during static postural tasks [103]. Similarly, in the StandTS task, stroke participants demonstrated significantly lower SOL/TA CoA on the CONTRA side compared to the healthy group, suggesting an impaired ability to coordinate eccentric and concentric muscle activity during transitional movements. These findings corroborate prior studies highlighting reduced neuromuscular efficiency in stroke survivors during tasks requiring controlled descent or weight transfer [1]. In contrast, during the GI task, stroke participants demonstrated elevated GM/TA CoA in the SWING limb compared to healthy participants. This increased coactivation likely represents a compensatory strategy to stabilise the limb during dynamic movement [21]. However, while functional in the short term, heightened CoA is associated with reduced movement efficiency and increased energy expenditure. Similar compensatory mechanisms have been widely reported in stroke populations, where antagonist activity increases to counteract instability during dynamic tasks [21,28].

Non-linear metrics offered a deeper perspective on the complexity and stability of CoA patterns. Across tasks, stroke participants consistently exhibited higher LyE and CI values compared to healthy individuals, indicating increased neuromuscular complexity and reduced stability. During the standing task, elevated LyE and CI values in the TA/SOL and Ve/Do muscle pairs indicate disorganised postural control and reduced stability. These results line up with previous research suggesting that increased variability in stroke survivors indicates rigid motor strategies and impaired adaptability, often stemming from disrupted sensory-motor integration [32]. In the StandTS task, increased complexity suggests that transitional movements demand greater neuromuscular coordination in stroke survivors, often resulting in inefficient and unstable control strategies. The higher LyE values observed on the CONTRA side further highlight its compensatory role during these tasks, consistent with findings that stroke survivors redistribute postural control to the CONTRA limb [3]. In the GI task, stroke participants demonstrated significantly elevated LyE and CI values in both the swing and stance limbs for SOL/TA and GM/TA muscle pairs. These findings indicate increased instability and a greater reliance on complex but disordered CoA strategies during dynamic movements. Elevated LyE values in the swing limb reflect reduced stability, while higher CI values suggest an over-reliance on redundant muscle activations, further supporting findings by [37].

In healthy individuals, CoA patterns were characterised by lower LyE and CI values across all tasks, reflecting stable and efficient neuromuscular control. Linear metrics also revealed consistent CoA between DOM and NDOM limbs, highlighting symmetrical and coordinated muscle activation during all tasks. These results align with previous studies emphasising the efficiency of neuromuscular systems in healthy populations, in which CoA is optimised for energy conservation and adaptability [4,24]. In contrast, the task-specific nature of CoA impairments in stroke participants underscores the influence of functional demands on neuromuscular control. During static tasks, reduced CoA during standing reflects a loss of postural control mechanisms, consistent with prior findings of diminished stability in stroke populations [37]. In transitional tasks, elevated CI during StandTS highlights the challenges stroke survivors face in coordinating dynamic weight transfers, which require precise modulation of antagonist activity [17,72]. For dynamic tasks, increased LyE and CI during GI illustrate inefficiencies in stabilising the limb during movement initiation, reflecting the compensatory strategies stroke survivors adopt to meet task demands [28]. These findings underscore the multifaceted nature of post-stroke CoA impairments, revealing that stroke participants struggle to adapt their neuromuscular strategies to the varying demands of different functional tasks.

### 4.7. Integration of Stiffness, COP Dynamics, and Antagonist Coactivation Patterns

The integration of stiffness metrics, COP dynamics, and CoA patterns in this study underlines the multifaceted nature of neuromuscular dysfunction and adaptation in stroke survivors, challenging traditional models of muscle tone assessment. The positive correlation between CONTRA iStiff and fStiff highlights the compensatory role of the CONTRA limb, suggesting that stiffness asymmetries play a critical role in post-stroke motor control. Conversely, the lack of consistent associations for the IPSI limb and the variability in task-specific outcomes underscore the limitations of relying solely on intrinsic and functional stiffness to fully characterise muscle tone. Impairments in COP dynamics further reveal significant deficits in postural complexity and dynamic stability, with stroke survivors relying on rigid and less adaptable strategies, particularly during tasks requiring APAs. These deficits align with the alterations in CoA, in which non-linear metrics provide critical insights into the neuromuscular complexity and inefficiencies that linear stiffness models cannot fully capture.

The findings of this study also have direct implications for rehabilitation strategies, particularly in developing targeted therapies to improve postural stability, neuromuscular control, and motor function in stroke survivors. Given the observed compensatory mechanisms in CoA and stiffness regulation, several rehabilitation approaches could leverage these insights, such as robotic-assisted training. Exoskeletons and robotic gait trainers can use muscle CoA patterns and stiffness data to personalise training intensity, ensuring task-specific modulation of muscle tone [122,123]. By integrating real-time stiffness and CoA metrics, these devices could provide adaptive resistance and support, encouraging active motor learning and reducing compensatory maladaptive strategies [123,124]. Virtual reality (VR)-based interventions can simulate real-life postural and balance challenges, allowing stroke survivors to practice neuromuscular control in safe, immersive settings [125]. Given that postural complexity and adaptability were impaired in stroke patients (as indicated by reduced LyE and COP dynamics), VR-based balance training could target these specific deficits by gradually increasing task complexity and environmental variability [126,127]. Functional electrical stimulation (FES) could be fine-tuned using non-linear COP dynamics and LyE values to enhance muscle coordination and balance recovery [128]. By modulating antagonist muscle CoA patterns, FES could reinforce more physiologically efficient movement patterns, mitigating excessive CoA reliance in dynamic tasks [128,129]. Also, the use of real-time CoA and COP feedback in rehabilitation settings could provide stroke survivors with quantifiable movement targets [130,131]. By teaching patients muscle activation levels in response to dynamic demands, biofeedback therapy could optimise postural adaptability and functional movement control [130,131].

These findings emphasise the importance of integrating biomechanics-based and neuromuscular models into rehabilitation frameworks [132,133]. By aligning rehabilitation protocols with task-specific CoA and postural stiffness modulation, clinicians could develop more targeted, data-driven therapies to improve stability, adaptability, and movement efficiency in stroke survivors, ultimately enhancing functional independence and quality of life [134].

### 4.8. Analysis with Existing Models of Postural Control

The findings of this study contribute to the ongoing discussion on PC mechanisms in stroke patients by providing a comparative analysis with existing biomechanical models. Traditional models, such as the inverted pendulum model, have been widely used to describe the balance and postural adjustments required to maintain stability [93]. While these models effectively capture the mechanical constraints of PC, they often fail to account for the neuromuscular complexity and adaptive control strategies employed by stroke survivors.

Recent studies have expanded on the inverted pendulum framework by integrating multi-segment models that account for segmental coordination, interlimb compensation, and neuromuscular feedback [12]. In contrast to these approaches, this study provides additional insights by incorporating non-linear analyses of CoA and COP dynamics, revealing task-specific compensatory mechanisms that are not captured by purely mechanical models. Our findings align with studies demonstrating that stroke survivors exhibit modified postural complexity and reduced adaptability, as reflected by decreased LyE values and COP variability during functional tasks [32].

Furthermore, biomechanical models that emphasise joint stiffness and muscle CoA as primary contributors to PC [135] suggest that increased stiffness may be a compensatory strategy in patients with motor impairments. Our results support this hypothesis, particularly in the CONTRA limb of stroke participants, where increased iStiff correlated with enhanced postural stability in standing tasks. However, the data also indicated that increased stiffness alone is insufficient for maintaining dynamic stability, as evidenced by the lack of consistent correlations with fStiff in more complex tasks like GI. This observation underscores the need for multi-factorial models that integrate biomechanical, neuromuscular, and sensory contributions to PC.

Additionally, while linear models have been useful in characterising postural deficits in stroke, this study highlights the limitations of relying solely on such approaches. Non-linear metrics such as LyE and COP MSE provide a more comprehensive view of postural adaptability, revealing the degree of neuromuscular complexity that traditional models may overlook [38]. Future studies should explore hybrid models that combine mechanical, neuromuscular, and computational frameworks to improve postural assessment and rehabilitation strategies for stroke survivors.

These findings suggest that while existing models have provided foundational insights into PC mechanisms, further refinements incorporating multi-segment analysis, non-linear dynamics, and machine learning-based predictions may enhance our ability to understand and optimise rehabilitation interventions for stroke survivors.

### 4.9. Advantages and Trade-offs of the Multidimensional Approach

A key contribution of this study is the integration of a multidimensional approach for evaluating postural tone, combining linear and non-linear analyses. This methodology provides a more comprehensive assessment of neuromuscular function compared to traditional single-metric evaluations, capturing both static and dynamic aspects of postural stability. However, as with any complex analytical approach, there are trade-offs that should be considered.

One primary advantage of this approach is its ability to detect subtle variations in PC that may be overlooked by single-metric assessments. Traditional linear measures, such as stiffness and dCOP, are effective in characterising gross motor deficits but fail to capture the temporal and adaptive components of neuromuscular control. The inclusion of non-linear metrics, such as LyE and MSE, allows for the assessment of neuromuscular adaptability and variability, which are critical for understanding motor impairments in stroke survivors [119,136].

Despite these advantages, integrating both linear and non-linear analyses comes with computational costs and practical challenges. Non-linear metrics typically require higher computational power and longer processing times compared to traditional assessments. For instance, calculating LyE involves embedding dynamic time-series data into high-dimensional spaces, which can be computationally intensive, especially when applied to large datasets [137]. This complexity may limit real-time applications in clinical settings, where rapid assessments are often preferred. However, advancements in automated data processing and machine learning algorithms could help mitigate these computational burdens in future implementations.

Another consideration is the interpretability of non-linear metrics. While they provide valuable insights into postural complexity and stability, their clinical applicability is still under investigation. Unlike linear measures, which have well-established normative ranges and clinical benchmarks, non-linear metrics require further validation to establish their utility in guiding rehabilitation interventions. Therefore, while this study highlights the potential of non-linear analyses, further research is needed to refine clinical interpretation and practical usability.

In summary, the multidimensional approach employed in this study offers a more holistic and detailed characterisation of postural tone but also presents trade-offs in terms of computational demands, interpretability, and clinical feasibility. Future studies should explore ways to optimise this methodology, such as developing streamlined computational models and integrating machine learning techniques to enhance real-time applicability in rehabilitation settings.

### 4.10. Considerations and Future Directions

While this study provides valuable insights into the neurobiomechanical underpinnings of postural tone, a few considerations should be acknowledged, particularly regarding sample size and longitudinal tracking. The relatively small sample size, especially in the stroke group, may limit the generalisability of the findings to the broader stroke population. Nevertheless, robust methodologies and the inclusion of both linear and non-linear analyses help mitigate this limitation by providing a comprehensive view of the observed patterns. Additionally, the heterogeneity of stroke participants, such as variations in lesion location, severity, and time since the event, reflects the real-world diversity of stroke populations, even though it may contribute to variability in the results. Incorporating a larger sample size across different stroke severity levels would allow for subgroup analyses, helping to identify whether different rehabilitation strategies are required based on stroke chronicity, lesion location, or mobility status. Another important factor to consider is the variability introduced by different stroke subtypes (ischemic vs. haemorrhagic), which may lead to distinct neuromuscular impairments and postural adaptations. Ischemic strokes, which account for the majority of cases, often result in motor impairments due to corticospinal damage, while haemorrhagic strokes may cause broader dysfunctions involving motor coordination and balance control [138]. Future studies should explore how these differences could influence postural tone regulation and whether rehabilitation strategies should be tailored based on stroke subtype.

Additionally, this study’s cross-sectional design offers a snapshot of neuromuscular dynamics but does not allow for tracking changes over time or in response to specific interventions. Stroke rehabilitation is a dynamic and evolving process, with neuromuscular adaptations occurring over weeks or months. Conducting longitudinal research could build on these findings to explore the progression of neuromuscular adaptations and the impact of targeted rehabilitation strategies. Furthermore, while the metrics used to quantify stiffness and coactivation provide critical insights, no single measurement seems to fully capture the complexity of postural tone. Future studies should incorporate longitudinal assessments to evaluate how muscle tone, stiffness, and coactivation patterns evolve throughout different stages of stroke recovery. This would help determine whether the observed postural control adaptations are stable over time or if they change in response to rehabilitation interventions. Also, while the tasks assessed are functionally relevant, individual variations in task execution, such as movement speed or strategy, may introduce some variability. Nevertheless, the inclusion of both static and dynamic tasks strengthens the ecological validity of the study. These considerations highlight opportunities for future research to further develop the multidimensional approach employed in this study, deepening our understanding of postural tone regulation and guiding more effective rehabilitation strategies.

This study could also benefit from exploring, in future research, the integration of wearable sensors and machine learning algorithms to enable real-time monitoring of postural dynamics, potentially facilitating personalised rehabilitation approaches. Real-time biofeedback systems, such as force plate monitoring, electromyography-driven stimulation, and augmented reality interfaces, could be incorporated into training programs to provide immediate adjustments in response to detected postural instability [139]. Such systems could improve functional outcomes by reinforcing adaptive postural control strategies, reducing reliance on compensatory mechanisms, and optimising motor learning.

The integration of advanced computational models, such as deep learning and transformer-based architectures, holds significant potential for enhancing the assessment of muscle tone and postural control. Recent studies have demonstrated the efficacy of transformer-based neural networks in predicting joint kinematics from sensor data, offering precise estimations of hip and knee joint angles during various activities [140]. Similarly, transformer models have been employed to decode electromyographic signals for hand gesture recognition, achieving high accuracy with reduced computational complexity [141].

In the context of PC, machine learning classifiers have been used to analyse kinematic data, facilitating the diagnosis of neurodegenerative disorders such as Alzheimer’s disease [142,143]. Additionally, transformer-based models have been applied to driver steering behaviour modelling, capturing complex neuromuscular patterns and informing the development of advanced driver-assistance systems [141].

These advances underscore the potential of integrating machine learning approaches with biomechanical models to enhance professionals’ understanding of neuromuscular control mechanisms. Future research could explore the application of transformer-based architectures to detect subtle neuromuscular patterns and improve classification models for post-stroke rehabilitation based on linear and non-linear variables. By leveraging large datasets and incorporating multimodal sensor inputs, such approaches could enhance the ability to characterise muscle tone dynamics and predict functional outcomes with greater accuracy.

While this study employs Spearman’s correlations and Mann-Whitney tests to analyse group differences and associations between key variables, we acknowledge that more advanced statistical techniques could further enhance the robustness of the findings. Multivariate regression models and machine learning approaches could offer deeper insights into the complex, non-linear relationships between biomechanical variables, allowing for improved classification of PC impairments in stroke survivors [144]. However, given the already extensive linear and non-linear biomechanical analyses of this study, implementing such models was beyond the scope of the current work.

Future research should explore multivariate statistical models, such as generalised estimating equations or mixed-effects models, to account for the potential influence of covariates such as stroke severity, lesion location, and rehabilitation history. Additionally, machine learning techniques, including support vector machines, random forests, and deep learning algorithms, could be leveraged to identify predictive markers of postural instability based on high-dimensional datasets. By incorporating such advanced methods in future studies, a more comprehensive and predictive framework for understanding postural tone regulation could be established, ultimately guiding the development of data-driven rehabilitation protocols tailored to individual stroke patients.

By addressing these limitations in future studies, a more comprehensive understanding of stroke-related postural dysfunctions can be achieved, allowing for the development of targeted and adaptive rehabilitation protocols that optimise motor recovery.

## 5. Conclusions

This study highlighted the multifaceted and complex nature of postural tone regulation by examining iStiff, fStiff, and CoA in healthy individuals and stroke survivors. The findings revealed significantly higher CONTRA iStiff and fStiff in stroke participants, reflecting the compensatory role of the CONTRA limb in maintaining stability. Conversely, the variability and lack of consistent associations in the IPSI limb underscored the complexity of neuromuscular adaptations post-stroke.

The analysis of CoA demonstrated that stroke participants exhibited reduced CoA in static and transitional tasks, such as standing and StandTS, while increased CoA was observed during dynamic tasks, such as GI. These results underscore the compensatory strategies employed by stroke participants to manage impaired motor control and stability demands.

Linear and non-linear analyses of COP and CoA dynamics showed distinct deficits in postural control among stroke participants. Stroke participants showed reduced aCOP and tdCOP during standing and altered pCOP during GI, indicating impaired stability and anticipatory adjustments. Non-linear metrics, such as CI and LyE, highlighted disorganised and inefficient control strategies, particularly during tasks requiring dynamic adjustments.

Together, these findings demonstrate that stroke survivors rely on compensatory mechanisms characterised by increased rigidity, reduced adaptability, and impaired stability to manage postural demands. The integration of linear and non-linear metrics provided deeper insights into these neuromuscular dysfunctions, highlighting the importance of multidimensional approaches to evaluating postural tone.

## Figures and Tables

**Table 1:** Electrode placement anatomical references [59,60].

Muscle	Anatomical Reference
Tibialis Anterior	Proximal third of the line between the tip of the fibula and the tip of the medial malleolus.
Soleus	2 cm distal to the lower border of the gastrocnemius medialis muscle belly and 2 cm medial to the posterior midline of the leg.
Gastrocnemius Medialis	Most prominent portion of muscle belly.

cm—centimetres.

**Table 2:** Antagonist coactivation formulae [21,99,102].

Functional Task	Muscle Pairs Identification	Antagonist Coactivation Formula (%)
Standing	TA/SOL	$\frac{{\mathrm{EMG}}_{\mathrm{TA}}}{{\mathrm{EMG}}_{\mathrm{SOL}}+{\mathrm{EMG}}_{\mathrm{TA}}}\;\times \;100$
	TA/GM	$\frac{{\mathrm{EMG}}_{\mathrm{TA}}}{{\mathrm{EMG}}_{\mathrm{GM}}+{\mathrm{EMG}}_{\mathrm{TA}}}\;\times \;100$
	Ve/Do	$\frac{{\mathrm{EMG}}_{\mathrm{TA}}}{{\mathrm{EMG}}_{\left(\mathrm{SOL}+\mathrm{GM}\right)}+{\mathrm{EMG}}_{\mathrm{TA}}}\;\times \;100$
Stand-to-Sit And Gait Initiation	SOL/TA	$\frac{{\mathrm{EMG}}_{\mathrm{SOL}}}{{\mathrm{EMG}}_{\mathrm{TA}}+{\mathrm{EMG}}_{\mathrm{SOL}}}\;\times \;100$
	GM/TA	$\frac{{\mathrm{EMG}}_{\mathrm{GM}}}{{\mathrm{EMG}}_{\mathrm{TA}}+{\mathrm{EMG}}_{\mathrm{GM}}}\;\times \;100$
	Do/Ve	$\frac{{\mathrm{EMG}}_{\left(\mathrm{SOL}+\mathrm{GM}\right)}}{{\mathrm{EMG}}_{\left(\mathrm{SOL}+\mathrm{TA}\right)}+{\mathrm{EMG}}_{\left(\mathrm{SOL}+\mathrm{GM}\right)}}\;\times \;100$

TA—tibialis anterior muscle; SOL—soleus muscle; GM—gastrocnemius medialis muscle; Do—dorsal muscles (SOL and GM); Ve—ventral muscles (TA); EMG—surface electromyographic activity.

**Table 3:** Participants’ characteristics: mean and standard deviation values for age, height, and weight of the healthy and stroke groups, as well as contralesional side, time since stroke for the stroke group, and dominance side for the healthy group.

	Mean (SD)		*p*-Value ^#^
	Healthy	Stroke	
Age (years)	46.42 (8.260)	48.50 (12.330)	0.699
Height (cm)	167.00 (12.000)	169.00 (8.000)	0.735
Weight (kg)	74.14 (12.550)	75.75 (12.520)	0.791
Sex	Female: n = 8	Female: n = 5	---
	Male: n = 4	Male: n = 7	
Contralesional Side	---	Left: n = 7	---
		Right: n = 5	
Dominant Side	Left: n = 1		
	Right: n = 11		
Time since stroke (months)	--	25.92 (21.470)	--

^#^ Student *t*-Test. SD—standard deviation; cm—centimetres; kg—kilograms.

**Table 4:** Median and 25th and 75th percentiles of intrinsic and functional stiffness in both healthy (dominant and non-dominant lower limb) and stroke (contralesional and ipsilesional lower limb) groups, and of antagonist coactivation of muscle pairs during standing and stand-to-sit (healthy: dominant and non-dominant lower limbs; stroke: contralesional and ipsilesional lower limbs), and during gait initiation (swing and stance lower limbs in both groups).

		Med (P25; P75)			
		Healthy		Stroke	
		DOM	NDOM	IPSI	CONTRA
iStiff (Nm/°)		0.30 (0.245; 0.407)	0.31 (0.212; 0.395)	0.81 (0.460; 1.740)	0.45 (0.333; 0.930)
fStiff (N/m)		10,157.00 (4212.453; 26,954.385)		3884.07 (1776.130; 19,366.177)	
Standing CoA (%)	TA/SOL	48.38 (43.973; 66.129)	55.49 (54.692; 70.726)	48.47 (25.690; 64.756)	28.78 (14.964; 41.062)
	TA/GM	42.64 (30.618; 64.032)	49.17 (24.527; 57.000)	45.46 (29.111; 61.551)	36.81 (30.159; 50.966)
	Ve/Do	68.58 (55.435; 75.260)	69.98 (61.364; 79.014)	59.27 (42.240; 82.229)	51.80 (31.741; 69.218)
StandTS CoA (%)	SOL/TA	47.53 (36.739; 65.565)	60.90 (48.341; 71.413)	40.50 (30.818; 50.821)	46.42 (32.534; 50.053)
	GM/TA	42.39 (35.478; 52.407)	40.05 (25.534; 66.400)	42.18 (17.791; 56.175)	48.44 (37.663; 50.935)
	Do/Ve	62.80 (52.914; 78.821)	73.63 (52.677; 82.664)	55.01 (46.826; 69.654)	64.04 (52.403; 67.065)
		SWING	STANCE	SWING	STANCE
GI CoA (%)	SOL/TA	54.08 (49.729; 69.836)	59.49 (38.588; 76.271)	50.85 (43.326; 54.262)	36.63 (29.798; 52.836)
	GM/TA	39.74 (25.409; 48.620)	52.76 (23.359; 78.350)	51.93 (40.917; 53.091)	34.80 (24.537; 44.198)
	Do/Ve	64.41 (60.298; 74.222)	76.00 (51.694; 89.011)	67.90 (65.953; 69.467)	53.43 (44.777; 60.817)

Med—median; P25—25th percentile; P75—75th percentile; N/m—Newton per meter; DOM—dominant lower limb; NDOM—non dominant lower limb; IPSI—ispilesional lower limb; CONTRA—contralesional lower limb; CoA—antagonist coactivation; TA—tibialis anterior; SOL—soleus; GM—gastrocnemius medialis; Ve—ventral muscles: tibialis anterior; Do—dorsal muscles: gastrocnemius medialis and soleus; Do—dorsal; Ve—ventral; SWING—first lower limb initiating gait; STANCE—second lower limb initiating gait.

**Table 5:** Spearman’s correlation analysis between intrinsic stiffness and functional stiffness variables.

	Correlated Variables		*r*	*p*-Value ^#^
A	iStiff DOM iStiff NDOM	fStiff HEALTHY	0.021 −0.133	0.948 0.680
B	iStiff IPSI iStiff CONTRA	fStiff STROKE	0.623 0.396	**0.030** 0.202
C	iStiff DOM iStiff NDOM	iStiff IPSI iStiff CONTRA	0.382 0.400	0.220 0.198
D	fStiff HEALTHY	fStiff STROKE	0.273	0.391

^#^ *p*-value for the Spearman correlation coefficient; significant differences are shown in bold. *r*—Spearman’s correlation coefficient; iStiff—intrinsic stiffness; fStiff—functional stiffness; DOM—dominant lower limb; NDOM—non-dominant lower limb; IPSI—ispilesional lower limb; CONTRA—contralesional lower limb.

**Table 6:** Spearman’s correlation analysis between intrinsic stiffness and antagonist coactivation values of different muscle pairs assessed during three functional tasks: standing, stand-to-sit, and gait initiation, in both stroke and healthy groups.

Task		CoA	*r*; *p* ^#^
Standing	HEALTHY iStiff DOM	TA/SOL DOM	0.448 *; **0.048**
		TA/GM DOM	0.039; 0.905
		Ve/Do DOM	0.340; 0.280
	HEALTHY iStiff NDOM	TA/SOL NDOM	0.446; 0.147
		TA/GM NDOM	0.168; 0.601
		Ve/Do NDOM	0.351; 0.263
	STROKE iStiff CONTRA	TA/SOL CONTRA	0.004; 0.991
		TA/GM CONTRA	0.618; 0.432
		Ve/Do CONTRA	0.246; 0.442
	STROKE iStiff IPSI	TA/SOL IPSI	0.294; 0.353
		TA/GM IPSI	0.308; 0.330
		Ve/Do IPSI	0.126; 0.696
StandTS	HEALTHY iStiff/DOM	SOL/TA DOM	0.004; 0.991
		GM/TA DOM	0.074; 0.820
		Do/Ve DOM	0.025; 0.940
	HEALTHY iStiff NDOM	SOL/TA NDOM	0.263; 0.409
		GM/TA NDOM	0.302; 0.340
		Do/Ve NDOM	0.168; 0.601
	STROKE iStiff CONTRA	SOL/TA CONTRA	0.681; 0.0150
		GM/TA CONTRA	0.446; 0.147
		Do/Ve CONTRA	0.403 *; **0.048**
	STROKE iStiff IPSI	SOL/TA IPSI	−0.165; 0.609
		GM/TA IPSI	−0.084; 0.795
		Do/Ve IPSI	−0.147; 0.648
Gait Initiation	HEALTHY iStiff SWING	SOL/TA SWING	−0.431; 0.162
		GM/TA SWING	0.410; 0.186
		Do/Ve SWING	−0.207; 0.519
	HEALTHY iStiff STANCE	SOL/TA STANCE	0.246; 0.442
		GM/TA STANCE	0.004; 0.991
		Do/Ve SYANCE	0.098; 0.761
	STROKE iStiff SWING	SOL/TA SWING	0.323; 0.306
		GM/TA SWING	0.060; 0.854
		Do/Ve SWING	−0.049; 0.879
	STROKE iStiff STANCE	SOL/TA STANCE	−0.221; 0.491
		GM/TA STANCE	0.035; 0.914
		Do/Ve STANCE	−0.196; 0.541

^#^ *p*-value for the Spearman correlation coefficient; significant differences are shown in bold. *r*—Spearman’s correlation coefficient; *—*r* value, when there are significant differences; iStiff—intrinsic stiffness; StandTS—stand-to-sit; GI—gait initiation; DOM—dominant lower limb; NDOM—non-dominant lower limb; IPSI—ispilesional lower limb; CONTRA—contralesional lower limb; SWING—first lower limb initiating gait; STANCE—second lower limb for gait; SOL—soleus; TA—tibialis anterior; GM—gastrocnemius medialis; Ve—ventral muscles; Do—dorsal muscles.

**Table 7:** Spearman’s correlation analysis between functional stiffness and the antagonist coactivation values of the different muscle pairs assessed during three functional tasks: standing, stand-to-sit, and gait initiation, in both stroke and healthy groups.

Task		CoA	*r*; *p* ^#^
Standing	HEALTHY fStiff	TA/SOL DOM	−0.434; 0.159
		TA/GM DOM	−0.21; 0.948
		Ve/Do DOM	−0.322; 0.308
		TA/SOL NDOM	−0.217; 0.499
		TA/GM NDOM	−0.245; 0.443
		Ve/Do NDOM	−0.231; 0.471
	STROKE fStiff	TA/SOL CONTRA	0.077; 0.812
		TA/GM CONTRA	0.350; 0.265
		Ve/Do CONTRA	0.217; 0.499
		TA/SOL IPSI	−0.140; 0.665
		TA/GM IPSI	−0.189; 0.557
		Ve/Do IPSI	−0.147; 0.649
StandTS	HEALTHY fStiff	SOL/TA DOM	0.175; 0.587
		GM/TA DOM	−0.049; 0.880
		Do/Ve DOM	−0.007; 0.983
		SOL/TA NDOM	−0.448; 0.145
		GM/TA NDOM	−0.434; 0.159
		Do/Ve NDOM	−0.427; 0.167
	STROKE fStiff	SOL/TA CONTRA	0.168; 0.602
		GM/TA CONTRA	0.266; 0.404
		Do/Ve CONTRA	0.098; 0.762
		SOL/TA IPSI	−0.112; 0.729
		GM/TA IPSI	−0.147; 0.649
		Do/Ve IPSI	−0.217; 0.499
Gait Initiation	HEALTHY fStiff	SOL/TA SWING	−0.147; 0.649
		GM/TA SWING	−0.770; 0.812
		Do/Ve SWING	−0.049; 0.880
		SOL/TA STANCE	−0.770; 0.812
		GM/TA STANCE	0.238; 0.457
		Do/Ve SYANCE	0.098; 0.762
	STROKE fStiff	SOL/TA SWING	0.098; 0.762
		GM/TA SWING	0.203; 0.527
		Do/Ve SWING	0.133; 0.681
		SOL/TA STANCE	0.420; 0.175
		GM/TA STANCE	0.587 *; **0.045**
		Do/Ve STANCE	0.434; 0.159

^#^ *p*-value for the Spearman correlation coefficient; significant differences are shown in bold. *r*—Spearman’s correlation coefficient; *—*r* value, when there are significant differences; fStiff—functional stiffness; StandTS—stand-to-sit; GI—gait initiation; DOM—dominant lower limb; NDOM—non-dominant lower limb; IPSI—ispilesional lower limb; CONTRA—contralesional lower limb; SWING—first lower limb initiating gait; STANCE—second lower limb for gait; SOL—soleus; TA—tibialis anterior; GM—gastrocnemius medialis; Ve—ventral muscles; Do—dorsal muscles.

**Table 8:** Spearman’s correlation analysis of antagonist coactivation between muscle pairs in the dominant and non-dominant lower limbs for standing and stand-to-sit, and between swing and stance limbs during gait initiation in the healthy group; and between the ipsilesional and contralesional lower limbs for standing and stand-to-sit, and between swing and stance limbs during gait initiation in the stroke group.

Task		CoA	*r*; *p* ^#^
Standing	HEALTHY DOM vs. NDOM	TA/SOL	0.832 *; **0.001**
		TA/GM	0.804 *; **0.002**
		Ve/Do	0.259; 0.417
	STROKE IPSI vs. CONTRA	TA/SOL	0.776 *; **0.003**
		TA/GM	0.699 *; **0.011**
		Ve/Do	0.315; 0.319
StandTS	HEALTHY DOM vs. NDOM	SOL/TA	0.573; 0.051
		GM/TA	0.762; **0.004**
		Do/Ve	0.573; 0.051
	STROKE IPSI vs. CONTRA	SOL/TA	0.231; 0.471
		GM/TA	0.007; 0.983
		Do/Ve	0.000; 1.000
Gait Initiation	HEALTHY SWING vs. STANCE	SOL/TA	0.364; 0.245
		GM/TA	−0.056; 0.863
		Do/Ve	0.231; 0.471
	STROKE SWING vs. STANCE	SOL/TA	0.112; 0.729
		GM/TA	0.483; 0.112
		Do/Ve	0.294; 0.354

^#^ *p*-value for the Spearman correlation coefficient; significant differences are shown in bold. r—Spearman’s correlation coefficient; *—*r* value, when there are significant differences; StandTS—stand-to-sit; GI—gait initiation; DOM—dominant lower limb; NDOM—non-dominant lower limb; IPSI—ispilesional lower limb; CONTRA—contralesional lower limb; SWING—first lower limb initiating gait; STANCE—second lower limb for gait; SOL—soleus; TA—tibialis anterior; GM—gastrocnemius medialis; Ve—ventral muscles; Do—dorsal muscles.

## Data Availability

Data are unavailable due to privacy or ethical restrictions.

## Abbreviations

The following abbreviations are used in this manuscript:

aCOP	COP amplitude
AP	Anteroposterior
CI	Complexity index
CNS	Central nervous system
CoA	Antagonist coactivation
COM	Centre of mass
CONTRA	Contralesional lower limb
COP	Centre of pressure
dCOP	COP displacement
DF	Dorsiflexion
Do	Dorsal muscles
DOM	Dominant lower limb
EMG	Electromyography
FES	Functional electrical stimulation
fStiff	Functional stiffness
GI	Gait initiation
GM	Gastrocnemius Medialis
GRF	Ground reaction forces
IPSI	Ipsilesional lower limb
iStiff	Intrinsic Stiffness
Kg	Kilograms
LyE	Lyapunov exponent
Med	Median
m	Metres
MSE	Multiscale Entropy
NDOM	Non-dominant lower limb
pCOP	COP position
PF	Plantar flexion
SD	Standard deviation
STANCE	Stance lower limb
StandTS	Stand-to-sit
SOL	Soleus
Swing	Swing lower limb
TA	Tibialis anterior
tdCOP	COP total displacement
uSt	Upright standing
Ve	Ventral muscles
VR	Virtual reality

## References

[1] Latash M.L., Huang X. (2015). Neural control of movement stability: Lessons from studies of neurological patients. Neuroscience.

[2] Profeta V.L.S., Turvey M.T. (2018). Bernstein’s levels of movement construction: A contemporary perspective. Hum. Mov. Sci..

[3] Ganguly J., Kulshreshtha D., Almotiri M., Jog M. (2021). Muscle Tone Physiology and Abnormalities. Toxins.

[4] Nielsen J.B., Christensen M.S., Farmer S.F., Lorentzen J. (2020). Spastic movement disorder: Should we forget hyperexcitable stretch reflexes and start talking about inappropriate prediction of sensory consequences of movement?. Exp. Brain Res..

[5] Latash M.L., Zatsiorsky V.M. (1993). Joint stiffness: Myth or reality?. Hum. Mov. Sci..

[6] Pandyan A.D., Hermens H.J., Conway B.A. (2016). Neurological Rehabilitation: Spasticity and Contractures in Clinical Practice and Research.

[7] Foerster O. (1936). The motor cortex in man in the light light of Hughlings Jackson’s Doctrines. Brain.

[8] Joshi V., Rouse E.J., Claflin E.S., Krishnan C. (2022). How Does Ankle Mechanical Stiffness Change as a Function of Muscle Activation in Standing and During the Late Stance of Walking?. IEEE Trans. Biomed. Eng..

[9] Sekiguchi Y., Muraki T., Owaki D., Honda K., Izumi S.I. (2018). Regulation of quasi-joint stiffness by combination of activation of ankle muscles in midstances during gait in patients with hemiparesis. Gait Posture.

[10] Mazlan M., Hamzah N., Ramakrishnan K. (2012). Unilateral ankle dorsiflexor spasticity: An uncommon, disabling complication of transverse myelitis. Arch. Med. Sci..

[11] Levin S.M. (2009). Human resting muscle tone (HRMT): Narrative, introduction and modern concepts [J. Bodywork Movement Ther. 12 (2008) 320–332]. J. Bodyw. Mov. Ther..

[12] Ivanenko Y., Gurfinkel V.S. (2018). Human Postural Control. Front. Neurosci..

[13] Graham J.V., Eustace C., Brock K., Swain E., Irwin-Carruthers S. (2009). The Bobath concept in contemporary clinical practice. Top. Stroke Rehabil..

[14] Shumway-Cook A., Woollacott M.H. (2017). Motor Control: Translating Research into Clinical Practice.

[15] Feldman A.G. (2016). The Relationship Between Postural and Movement Stability. Adv. Exp. Med. Biol..

[16] Sousa A.S., Macedo R., Santos R., Sousa F., Silva A., Tavares J.M. (2016). Influence of prolonged wearing of unstable shoes on upright standing postural control. Hum. Mov. Sci..

[17] Sousa A.S., Silva A., Santos R. (2015). Ankle anticipatory postural adjustments during gait initiation in healthy and post-stroke subjects. Clin. Biomech..

[18] Silva A., Sousa A.S., Pinheiro R., Ferraz J., Tavares J.M., Santos R., Sousa F. (2013). Activation timing of soleus and tibialis anterior muscles during sit-to-stand and stand-to-sit in post-stroke vs. healthy subjects. Somatosens. Mot. Res..

[19] Silva A., Sousa A.S., Pinheiro R., Tavares J.M., Santos R., Sousa F. (2012). Soleus activity in post-stroke subjects: Movement sequence from standing to sitting. Somatosens. Mot. Res..

[20] Boudarham J., Hameau S., Zory R., Hardy A., Bensmail D., Roche N. (2016). Coactivation of Lower Limb Muscles during Gait in Patients with Multiple Sclerosis. PLoS ONE.

[21] Pinho L., Sousa A.S.P., Silva C., Cunha C., Santos R., Tavares J.M.R.S., Pereira S., Pinheiro A.R., Félix J., Pinho F. (2023). Antagonist Coactivation of Muscles of Ankle and Thigh in Post-Stroke vs. Healthy Subjects during Sit-to-Stand Task. Appl. Sci..

[22] Chambers V., Artemiadis P. (2023). Using robot-assisted stiffness perturbations to evoke aftereffects useful to post-stroke gait rehabilitation. Front. Robot. AI.

[23] Paci M. (2003). Physiotherapy based on the Bobath concept for adults with post-stroke hemiplegia: A review of effectiveness studies. J. Rehabil. Med..

[24] Ruhe A., Fejer R., Walker B. (2010). The test-retest reliability of centre of pressure measures in bipedal static task conditions—A systematic review of the literature. Gait Posture.

[25] Latash M.L. (2018). Stability of Kinesthetic Perception in Efferent-Afferent Spaces: The Concept of Iso-perceptual Manifold. Neuroscience.

[26] Latash M.L., Zatsiorsky V. (2015). Biomechanics and Motor Control: Defining Central Concepts.

[27] Farinelli V., Bolzoni F., Marchese S.M., Esposti R., Cavallari P. (2021). A Novel Viewpoint on the Anticipatory Postural Adjustments During Gait Initiation. Front. Hum. Neurosci..

[28] Pilla A., Trigili E., McKinney Z., Fanciullacci C., Malasoma C., Posteraro F., Crea S., Vitiello N. (2020). Robotic Rehabilitation and Multimodal Instrumented Assessment of Post-stroke Elbow Motor Functions—A Randomized Controlled Trial Protocol. Front. Neurol..

[29] Lee S., Jakubowski K., Spear S., Rymer W. (2019). Muscle material properties in passive and active stroke-impaired muscle. J. Biomech..

[30] Xie T., Leng Y., Zhi Y., Jiang C., Tian N., Luo Z., Yu H., Song R. (2020). Increased Muscle Activity Accompanying With Decreased Complexity as Spasticity Appears: High-Density EMG-Based Case Studies on Stroke Patients. Front. Bioeng. Biotechnol..

[31] Tigrini A., Verdini F., Fioretti S., Mengarelli A. (2022). Long term correlation and inhomogeneity of the inverted pendulum sway time-series under the intermittent control paradigm. Commun. Nonlinear Sci. Numer. Simul..

[32] Stergiou N., Decker L.M. (2011). Human movement variability, nonlinear dynamics, and pathology: Is there a connection?. Hum. Mov. Sci..

[33] Suzuki Y., Nakamura A., Milosevic M., Nomura K., Tanahashi T., Endo T., Sakoda S., Morasso P., Nomura T. (2020). Postural instability via a loss of intermittent control in elderly and patients with Parkinson’s disease: A model-based and data-driven approach. Chaos.

[34] Freitas M., Pinho F., Pinho L., Silva S., Figueira V., Vilas-Boas J.P., Silva A. (2024). Biomechanical Assessment Methods Used in Chronic Stroke: A Scoping Review of Non-Linear Approaches. Sensors.

[35] Amirpourabasi A., Lamb S.E., Chow J.Y., Williams G.K.R. (2022). Nonlinear Dynamic Measures of Walking in Healthy Older Adults: A Systematic Scoping Review. Sensors.

[36] Kędziorek J., Błażkiewicz M. (2020). Nonlinear Measures to Evaluate Upright Postural Stability: A Systematic Review. Entropy.

[37] Stergiou N. (2016). Nonlinear Analysis for Human Movement Variability.

[38] Mengarelli A., Tigrini A., Verdini F., Rabini R., Fioretti S. (2023). Multiscale Fuzzy Entropy Analysis of Balance: Evidences of Scale-Dependent Dynamics on Diabetic Patients With and Without Neuropathy. IEEE Trans. Neural Syst. Rehabil. Eng..

[39] Caballero C., Barbado D., Moreno F. (2014). Non-linear tools and methodological concerns measuring human movement variability: Anoverview. Eur. J. Hum. Mov..

[40] Harbourne R.T., Stergiou N. (2009). Movement variability and the use of nonlinear tools: Principles to guide physical therapist practice. Phys. Ther..

[41] Lamontagne A., Richards C.L., Malouin F. (2000). Coactivation during gait as an adaptive behavior after stroke. J. Electromyogr. Kinesiol..

[42] Lamontagne A., Malouin F., Richards C.L., Dumas F. (2002). Mechanisms of disturbed motor control in ankle weakness during gait after stroke. Gait Posture.

[43] Sousa A.S.P., Silva A., Santos R., Sousa F., Tavares J.M.R.S. (2013). Interlimb Coordination During the Stance Phase of Gait in Subjects With Stroke. Arch. Phys. Med. Rehabil..

[44] Bohannon R.W., Smith M.B. (1987). Interrater reliability of a modified Ashworth scale of muscle spasticity. Phys. Ther..

[45] Dick J.P., Guiloff R.J., Stewart A., Blackstock J., Bielawska C., Paul E.A., Marsden C.D. (1984). Mini-mental state examination in neurological patients. J. Neurol. Neurosurg. Psychiatry.

[46] Sousa A.S.P., Santos R., Oliveira F.P.M., Carvalho P., Tavares J.M.R.S. (2012). Analysis of ground reaction force and electromyographic activity of the gastrocnemius muscle during double support. Proc. Inst. Mech.Eng. Part. H J. Eng. Med..

[47] Thompson P.D., Arena R., Riebe D., Pescatello L.S. (2013). ACSM’s new preparticipation health screening recommendations from ACSM’s guidelines for exercise testing and prescription, ninth edition. Curr. Sports Med. Rep..

[48] Cruz J., Jácome C., Oliveira A., Paixão C., Rebelo P., Flora S., Januário F., Valente C., Andrade L., Marques A. (2021). Construct validity of the brief physical activity assessment tool for clinical use in COPD. Clin. Respir. J..

[49] Marshall A.L., Smith B.J., Bauman A.E., Kaur S. (2005). Reliability and validity of a brief physical activity assessment for use by family doctors. Br. J. Sports Med..

[50] Folstein M.F., Folstein S.E., McHugh P.R. (1975). “Mini-mental state”. A practical method for grading the cognitive state of patients for the clinician. J. Psychiatr. Res..

[51] Guerreiro M., Silva A., Botelho M., Leitão O., Castro-Caldas A., Garcia C., Botelho M., Guerreiro M., Silva A., Botelho A. (1994). Adaptação à população portuguesa da tradução do Mini Mental State Examination. Rev. Port. Neurol..

[52] Guerreiro M. (1998). Contributo da Neuropsicologia para o Estudo das Demências. Ph.D. Thesis.

[53] Ansari N.N., Naghdi S., Arab T.K., Jalaie S. (2008). The interrater and intrarater reliability of the Modified Ashworth Scale in the assessment of muscle spasticity: Limb and muscle group effect. NeuroRehabilitation.

[54] Costa S.V. (2003). Adaptação e Validação Cultural e Linguıstica do Fugl-Meyer Assessment of Sensorimotor Recovery After Stroke.

[55] Santos A.P., Ramos N.C., Estêvão P.C., Lopes A., Pascoalinho J. (2005). Instrumentos de medida uteis no contexto da avaliação em fisioterapia. Re (Habilitar)-Rev. ESSA.

[56] Fugl-Meyer A.R., Jääskö L., Leyman I., Olsson S., Steglind S. (1975). The post-stroke hemiplegic patient. 1. a method for evaluation of physical performance. Scand. J. Rehabil. Med..

[57] Sanford J., Moreland J., Swanson L.R., Stratford P.W., Gowland C. (1993). Reliability of the Fugl-Meyer assessment for testing motor performance in patients following stroke. Phys. Ther..

[58] Drouin J.M., Valovich-mcLeod T.C., Shultz S.J., Gansneder B.M., Perrin D.H. (2004). Reliability and validity of the Biodex system 3 pro isokinetic dynamometer velocity, torque and position measurements. Eur. J. Appl. Physiol..

[59] Hermens H.J., Freriks B., Disselhorst-Klug C., Rau G. (2000). Development of recommendations for SEMG sensors and sensor placement procedures. J. Electromyogr. Kinesiol..

[60] Couto A.G.B., Vaz M.A.P., Pinho L., Felix J., Moreira J., Pinho F., Mesquita I.A., Montes A.M., Crasto C., Sousa A.S.P. (2023). Repeatability and Temporal Consistency of Lower Limb Biomechanical Variables Expressing Interlimb Coordination during the Double-Support Phase in People with and without Stroke Sequelae. Sensors.

[61] Hoang P.D., Gorman R.B., Todd G., Gandevia S.C., Herbert R.D. (2005). A new method for measuring passive length-tension properties of human gastrocnemius muscle in vivo. J. Biomech..

[62] Hufschmidt A., Mauritz K.H. (1985). Chronic transformation of muscle in spasticity: A peripheral contribution to increased tone. J. Neurol. Neurosurg. Psychiatry.

[63] Lakie M., Robson L.G. (1988). Thixotropic changes in human muscle stiffness and the effects of fatigue. Q. J. Exp. Physiol..

[64] Taylor D.C., Dalton J.D., Seaber A.V., Garrett W.E. (1990). Viscoelastic properties of muscle-tendon units. The biomechanical effects of stretching. Am. J. Sports Med..

[65] Sousa A.S., Tavares J.M. (2015). Interlimb Coordination During Step-to-Step Transition and Gait Performance. J. Mot. Behav..

[66] Le Clair K., Riach C. (1996). Postural stability measures: What to measure and for how long. Clin. Biomech..

[67] Camargos A.C., Rodrigues-de-Paula-Goulart F., Teixeira-Salmela L.F. (2009). The effects of foot position on the performance of the sit-to-stand movement with chronic stroke subjects. Arch. Phys. Med. Rehabil..

[68] Pinsault N., Vuillerme N. (2009). Test-retest reliability of centre of foot pressure measures to assess postural control during unperturbed stance. Med. Eng. Phys..

[69] Zok M., Mazzà C., Cappozzo A. (2008). Should the instructions issued to the subject in traditional static posturography be standardised?. Med. Eng. Phys..

[70] Aryan R., Inness E., Patterson K.K., Mochizuki G., Mansfield A. (2023). Reliability of force plate-based measures of standing balance in the sub-acute stage of post-stroke recovery. Heliyon.

[71] Kitabayashi T., Demura S., Noda M. (2003). Examination of the factor structure of center of foot pressure movement and cross-validity. J. Physiol. Anthropol. Appl. Hum. Sci..

[72] Silva A., Sousa A.S., Silva C., Tavares J.M., Santos R., Sousa F. (2015). Ankle antagonist coactivation in the double-support phase of walking: Stroke vs. healthy subjects. Somatosens. Mot. Res..

[73] Cheng P.T., Chen C.L., Wang C.M., Hong W.H. (2004). Leg muscle activation patterns of sit-to-stand movement in stroke patients. Am. J. Phys. Med. Rehabil..

[74] Dubost V., Beauchet O., Manckoundia P., Herrmann F., Mourey F. (2005). Decreased trunk angular displacement during sitting down: An early feature of aging. Phys. Ther..

[75] Inkster L.M., Eng J.J. (2004). Postural control during a sit-to-stand task in individuals with mild Parkinson’s disease. Exp. Brain Res..

[76] Arsenault A.B., Winter D.A., Marteniuk R.G., Hayes K.C. (1986). How many strides are required for the analysis of electromyographic data in gait?. Scand. J. Rehabil. Med..

[77] Fernandes Â., Sousa A.S.P., Rocha N., Tavares J.M.R.S. (2017). The Influence of a Cognitive Task on the Postural Phase of Gait Initiation in Parkinson’s Disease: An Electromyographic-Based Analysis. Mot. Control.

[78] Mann R.A., Hagy J.L., White V., Liddell D. (1979). The initiation of gait. J. Bone Jt. Surg. Am..

[79] Miller C.A., Verstraete M.C. (1996). Determination of the step duration of gait initiation using a mechanical energy analysis. J. Biomech..

[80] Burnett D.R., Campbell-Kyureghyan N.H., Cerrito P.B., Quesada P.M. (2011). Symmetry of ground reaction forces and muscle activity in asymptomatic subjects during walking, sit-to-stand, and stand-to-sit tasks. J. Electromyogr. Kinesiol..

[81] Caderby T., Yiou E., Peyrot N., de Viviés X., Bonazzi B., Dalleau G. (2017). Effects of Changing Body Weight Distribution on Mediolateral Stability Control during Gait Initiation. Front. Hum. Neurosci..

[82] Rajachandrakumar R., Fraser J.E., Schinkel-Ivy A., Inness E.L., Biasin L., Brunton K., McIlroy W.E., Mansfield A. (2017). Atypical anticipatory postural adjustments during gait initiation among individuals with sub-acute stroke. Gait Posture.

[83] Osada Y., Motojima N., Kobayashi Y., Yamamoto S. (2022). Differences in mediolateral dynamic stability during gait initiation according to whether the non-paretic or paretic leg is used as the leading limb. PLoS ONE.

[84] Basmajian J.V., De Luca C.J. (1985). Muscles Alive: Their Functions Revealed by Electromyography.

[85] Correia P.P., Mil-Homens P., FMH EDIÇÕES (2004). A Electromiografia no Estudo do Movimento Humano.

[86] Gajdosik R.L., Vander Linden D.W., Williams A.K. (1996). Influence of age on concentric isokinetic torque and passive extensibility variables of the calf muscles of women. Eur. J. Appl. Physiol. Occup. Physiol..

[87] Lamontagne A., Malouin F., Richards C.L. (2000). Contribution of passive stiffness to ankle plantarflexor moment during gait after stroke. Arch. Phys. Med. Rehabil..

[88] Riemann B.L., DeMont R.G., Ryu K., Lephart S.M. (2001). The Effects of Sex, Joint Angle, and the Gastrocnemius Muscle on Passive Ankle Joint Complex Stiffness. J. Athl. Train..

[89] Criswell E. (2010). Cram’s Introduction to Surface Electromyography.

[90] Nordez A., Cornu C., McNair P. (2006). Acute effects of static stretching on passive stiffness of the hamstring muscles calculated using different mathematical models. Clin. Biomech..

[91] Sousa A.S.P., Santos R., Silva A. (2017). Ankle Intrinsic Stiffness in Subcortical Poststroke Subjects. J. Mot. Behav..

[92] Salsich G.B., Mueller M.J., Sahrmann S.A. (2000). Passive ankle stiffness in subjects with diabetes and peripheral neuropathy versus an age-matched comparison group. Phys. Ther..

[93] Winter D.A. (1995). Human balance and posture control during standing and walking. Gait Posture.

[94] Winter D.A., Patla A.E., Prince F., Ishac M., Gielo-Perczak K. (1998). Stiffness control of balance in quiet standing. J. Neurophysiol..

[95] Winter D.A., Patla A.E., Rietdyk S., Ishac M.G. (2001). Ankle muscle stiffness in the control of balance during quiet standing. J. Neurophysiol..

[96] Carver T., Nadeau S., Leroux A. (2011). Relation between physical exertion and postural stability in hemiparetic participants secondary to stroke. Gait Posture.

[97] Fernandes Â., Sousa A.S., Couras J., Rocha N., Tavares J.M. (2015). Influence of dual-task on sit-to-stand-to-sit postural control in Parkinson’s disease. Med. Eng. Phys..

[98] Mizusawa H., Jono Y., Iwata Y., Kinoshita A., Hiraoka K. (2017). Processes of anticipatory postural adjustment and step movement of gait initiation. Hum. Mov. Sci..

[99] Kellis E., Arabatzi F., Papadopoulos C. (2003). Muscle co-activation around the knee in drop jumping using the co-contraction index. J. Electromyogr. Kinesiol..

[100] Persaud R. (1994). Correlation, regression, and repeated data. BMJ.

[101] Crenna P., Cuong D.M., Brénière Y. (2001). Motor programmes for the termination of gait in humans: Organisation and velocity-dependent adaptation. J. Physiol..

[102] Falconer K., Winter D.A. (1985). Quantitative assessment of co-contraction at the ankle joint in walking. Electromyogr. Clin. Neurophysiol..

[103] Kesar T.M., Tan A., Eicholtz S., Baker K., Xu J., Anderson J.T., Wolf S.L., Borich M.R. (2019). Agonist-Antagonist Coactivation Enhances Corticomotor Excitability of Ankle Muscles. Neural Plast.

[104] Costa M., Goldberger A.L., Peng C.K. (2002). Multiscale entropy analysis of complex physiologic time series. Phys. Rev. Lett..

[105] Busa M.A., van Emmerik R.E.A. (2016). Multiscale entropy: A tool for understanding the complexity of postural control. J. Sport Health Sci..

[106] Busa M.A., Jones S.L., Hamill J., van Emmerik R.E.A. (2016). Multiscale entropy identifies differences in complexity in postural control in women with multiple sclerosis. Gait Posture.

[107] Costa M., Goldberger A.L., Peng C.K. (2005). Multiscale entropy analysis of biological signals. Phys. Rev. E-Stat. Nonlinear Soft Matter Phys..

[108] Goldberger A.L., Amaral L.A., Glass L., Hausdorff J.M., Ivanov P.C., Mark R.G., Mietus J.E., Moody G.B., Peng C.K., Stanley H.E. (2000). PhysioBank, PhysioToolkit, and PhysioNet: Components of a new research resource for complex physiologic signals. Circulation.

[109] Richman J.S., Moorman J.R. (2000). Physiological time-series analysis using approximate entropy and sample entropy. Am. J. Physiol. Heart Circ. Physiol..

[110] Caesarendra W., Kosasih B., Tieu K., Moodie C.A. An application of nonlinear feature extraction-A case study for low speed slewing bearing condition monitoring and prognosis. Proceedings of the 2013 IEEE/ASME International Conference on Advanced Intelligent Mechatronics.

[111] Rosenstein M.T., Collins J.J., De Luca C.J. (1993). A practical method for calculating largest Lyapunov exponents from small data sets. Phys. D Nonlinear Phenom..

[112] Broomhead D.S., King G.P. (1986). Extracting qualitative dynamics from experimental data. Phys. D Nonlinear Phenom..

[113] Matilla-García M., Morales I., Rodríguez J.M., Ruiz Marín M. (2021). Selection of Embedding Dimension and Delay Time in Phase Space Reconstruction via Symbolic Dynamics. Entropy.

[114] Vlachos I., Kugiumtzis D. (2008). State Space Reconstruction for Multivariate Time Series Prediction. arXiv.

[115] Costa M.D., Goldberger A.L. (2015). Generalized Multiscale Entropy Analysis: Application to Quantifying the Complex Volatility of Human Heartbeat Time Series. Entropy.

[116] Mademli L., Mavridi D., Bohm S., Patikas D.A., Santuz A., Arampatzis A. (2021). Standing on unstable surface challenges postural control of tracking tasks and modulates neuromuscular adjustments specific to task complexity. Sci. Rep..

[117] Marouvo J., Sousa F., Fernandes O., Castro M.A., Paszkiel S. (2021). Gait Kinematics Analysis of Flatfoot Adults. Appl. Sci..

[118] Marôco J. (2018). Análise Estatística com o SPSS Statistics.

[119] Sainburg R., Good D., Przybyla A. (2013). Bilateral Synergy: A Framework for Post-Stroke Rehabilitation. J. Neurol Transl. Neurosci..

[120] Pavol M.J., Owings T.M., Foley K.T., Grabiner M.D. (2001). Mechanisms leading to a fall from an induced trip in healthy older adults. J. Gerontol. A Biol. Sci. Med. Sci..

[121] Mehdizadeh H., Khalaf K., Ghomashchi H., Taghizadeh G., Ebrahimi I., Taghavi Azar Sharabiani P., Mousavi S.J., Parnianpour M. (2018). Effects of cognitive load on the amount and temporal structure of postural sway variability in stroke survivors. Exp. Brain Res..

[122] Mehrholz J., Thomas S., Kugler J., Pohl M., Elsner B. (2020). Electromechanical-assisted training for walking after stroke. Cochrane Database Syst. Rev..

[123] Wen S., Huang R., Liu L., Zheng Y., Yu H. (2024). Robotic exoskeleton-assisted walking rehabilitation for stroke patients: A bibliometric and visual analysis. Front. Bioeng. Biotechnol..

[124] Jixian W., Yongfang L., Lin Q., Muyassar M., Chang L., Ze L., Rubing S., Shengju W., Guo-Yuan Y. (2024). Advanced rehabilitation in ischaemic stroke research. Stroke Vasc. Neurol..

[125] Cano-de-la-Cuerda R., Blázquez-Fernández A., Marcos-Antón S., Sánchez-Herrera-Baeza P., Fernández-González P., Collado-Vázquez S., Jiménez-Antona C., Laguarta-Val S. (2024). Economic Cost of Rehabilitation with Robotic and Virtual Reality Systems in People with Neurological Disorders: A Systematic Review. J. Clin. Med..

[126] Laver K.E., Lange B., George S., Deutsch J.E., Saposnik G., Crotty M. (2017). Virtual reality for stroke rehabilitation. Cochrane Database Syst. Rev..

[127] Khan A., Imam Y.Z., Muneer M., Al Jerdi S., Gill S.K. (2024). Virtual reality in stroke recovery: A meta-review of systematic reviews. Bioelectron. Med..

[128] Kristensen M.G.H., Busk H., Wienecke T. (2022). Neuromuscular Electrical Stimulation Improves Activities of Daily Living Post Stroke: A Systematic Review and Meta-analysis. Arch. Rehabil. Res. Clin. Transl..

[129] Huber J., Kaczmarek K., Leszczyńska K., Daroszewski P. (2022). Post-Stroke Treatment with Neuromuscular Functional Electrostimulation of Antagonistic Muscles and Kinesiotherapy Evaluated with Electromyography and Clinical Studies in a Two-Month Follow-Up. Int. J. Environ. Res. Public Health.

[130] Hinton E.H., Buffum R., Kingston D., Stergiou N., Kesar T., Bierner S., Knarr B.A. (2024). Real-Time Visual Kinematic Feedback During Overground Walking Improves Gait Biomechanics in Individuals Post-Stroke. Ann. Biomed. Eng..

[131] Levin M.F., and Demers M. (2021). Motor learning in neurological rehabilitation. Disabil. Rehabil..

[132] Campagnini S., Arienti C., Patrini M., Liuzzi P., Mannini A., Carrozza M.C. (2022). Machine learning methods for functional recovery prediction and prognosis in post-stroke rehabilitation: A systematic review. J. NeuroEng. Rehabil..

[133] Boukhennoufa I., Zhai X., Utti V., Jackson J., McDonald-Maier K.D. (2022). Wearable sensors and machine learning in post-stroke rehabilitation assessment: A systematic review. Biomed. Signal Process. Control.

[134] Sengupta N., Rao A.S., Yan B., Palaniswami M. (2024). A Survey of Wearable Sensors and Machine Learning Algorithms for Automated Stroke Rehabilitation. IEEE Access.

[135] Loram I.D., Lakie M. (2002). Direct measurement of human ankle stiffness during quiet standing: The intrinsic mechanical stiffness is insufficient for stability. J. Physiol..

[136] Liu J., Wang J., Tan G., Sheng Y., Chang H., Xie Q., Liu H. (2021). Correlation Evaluation of Functional Corticomuscular Coupling With Abnormal Muscle Synergy After Stroke. IEEE Trans. Biomed. Eng..

[137] Hussain V.S., Spano M.L., Lockhart T.E. (2020). Effect of data length on time delay and embedding dimension for calculating the Lyapunov exponent in walking. J. R. Soc. Interface.

[138] Kwakkel G., Kollen B., Lindeman E. (2004). Understanding the pattern of functional recovery after stroke: Facts and theories. Restor. Neurol. Neurosci..

[139] Kosonogov V., Medvedeva A., Komilova F., Volodina M. (2024). Postural control in emotional states: An effect of biofeedback. Gait Posture.

[140] Ren J., Wang A., Li H., Yue X., Meng L. (2023). A Transformer-Based Neural Network for Gait Prediction in Lower Limb Exoskeleton Robots Using Plantar Force. Sensors.

[141] Mahboob T., Chung M.Y., Choi K.W. (2023). EMG-based 3D hand gesture prediction using transformer–encoder classification. ICT Express.

[142] Costa L., Gago M.F., Yelshyna D., Ferreira J., David Silva H., Rocha L., Sousa N., Bicho E. (2016). Application of Machine Learning in Postural Control Kinematics for the Diagnosis of Alzheimer’s Disease. Comput. Intell. Neurosci..

[143] Choo Y.J., Chang M.C. (2022). Use of Machine Learning in Stroke Rehabilitation: A Narrative Review. Brain Neurorehabil.

[144] Bennett T., Kumar P., Garate V.R. (2022). A Machine Learning Model for Predicting Sit-to-Stand Trajectories of People with and without Stroke: Towards Adaptive Robotic Assistance. Sensors.

